# Requirement for Pdx1 in specification of latent endocrine progenitors in zebrafish

**DOI:** 10.1186/1741-7007-9-75

**Published:** 2011-10-31

**Authors:** Robin A Kimmel, Lucas Onder, Armin Wilfinger, Elin Ellertsdottir, Dirk Meyer

**Affiliations:** 1 Institute of Molecular Biology/CMBI; Leopold-Francis University, Technikerstrasse 25, A-6020 Innsbruck, Austria; 2 Institute of Immunobiology, LFA, Kantonsspital St. Gallen, Rorschacherstrasse 95, CH-6900 St. Gallen, Switzerland; 3 Growth and Development, Biozentrum, University of Basel, Klingelbergstrasse 50/70, CH-4056 Basel, Switzerland

## Abstract

**Background:**

Insulin-producing beta cells emerge during pancreas development in two sequential waves. Recently described later-forming beta cells in zebrafish show high similarity to second wave mammalian beta cells in developmental capacity. Loss-of-function studies in mouse and zebrafish demonstrated that the homeobox transcription factors Pdx1 and Hb9 are both critical for pancreas and beta cell development and discrete stage-specific requirements for these genes have been uncovered. Previously, exocrine and endocrine cell recovery was shown to follow loss of *pdx1 *in zebrafish, but the progenitor cells and molecular mechanisms responsible have not been clearly defined. In addition, interactions of *pdx1 *and *hb9 *in beta cell formation have not been addressed.

**Results:**

To learn more about endocrine progenitor specification, we examined beta cell formation following morpholino-mediated depletion of *pdx1 *and *hb9*. We find that after early beta cell reduction, recovery occurs following loss of either *pdx1 *or *hb9 *function. Unexpectedly, simultaneous knockdown of both *hb9 *and *pdx1 *leads to virtually complete and persistent beta cell deficiency. We used a *NeuroD:EGFP *transgenic line to examine endocrine cell behavior *in vivo *and developed a novel live-imaging technique to document emergence and migration of late-forming endocrine precursors in real time. Our data show that Notch-responsive progenitors for late-arising endocrine cells are predominantly post mitotic and depend on *pdx1*. By contrast, early-arising endocrine cells are specified and differentiate independent of *pdx1*.

**Conclusions:**

The nearly complete beta cell deficiency after combined loss of *hb9 *and *pdx1 *suggests functional cooperation, which we clarify as distinct roles in early and late endocrine cell formation. A novel imaging approach permitted visualization of the emergence of late endocrine cells within developing embryos for the first time. We demonstrate a *pdx1*-dependent progenitor population essential for the formation of duct-associated, second wave endocrine cells. We further reveal an unexpectedly low mitotic activity in these progenitor cells, indicating that they are set aside early in development.

## Background

In the vertebrate pancreas, the increase in endocrine cells during late and post-embryonic development is caused by *de novo *progenitor differentiation and proliferation of pre-existing endocrine cells [1]. In mice, the majority of endocrine cells arise after embryonic day 12.5 (e12.5), from cells located in the branched epithelium of the dorsal and ventral buds. These second wave endocrine cells delaminate, migrate and cluster into numerous islets [1,2]. In addition, early-forming, first wave endocrine cells of unknown function appear before epithelial branching at e9.5, but do not contribute to mature islets [1]. Only recently, two waves of endocrine cell development were also observed in zebrafish. Similarly, cells with a mature gene expression profile only form during the second wave, while the early cells do not contribute to the later bulk of mature beta cells that produce insulin at high levels [3].

The zebrafish pancreas arises from two progenitor domains called the dorsal bud and ventral bud (DB and VB, respectively), which have distinct differentiation potentials [3,4]. The DB forms before 24 h post fertilization (hpf), and consists of clustered early endocrine cells called the principal islet. The VB arises from the gut tube after 34 hpf, and these cells migrate to engulf the principal islet [4,5]. Cells of the VB expand posteriorly to form the pancreatic tail and differentiate into exocrine cells, duct cells, and late arising (second wave) endocrine cells. The ventral bud-derived ductal system includes the attachment of the pancreas to the gut (extrapancreatic duct (EPD)), and a branching network in the expanding pancreatic tail (intrapancreatic duct (IPD)). Late endocrine cells in zebrafish appear to originate from progenitors located in or adjacent to the ductal system and contribute to expansion of the large principal islet and also coalesce to form scattered smaller secondary islets [5-8]. The emergence of late endocrine cells is considered analogous to the second wave of endocrine cell differentiation in mammals [3,5,7].

Pancreas formation requires the function of a highly conserved network of hierarchically expressed transcription factors. Among them, Pdx1 and Hb9 (also called Mnx1, Hlxb9) play key roles in pancreas and beta cell development, as demonstrated by loss-of-function studies in mice [9,10]. *Pdx1 *is expressed throughout the early pancreatic progenitor domain and is highly expressed in mature beta cells. Pdx1 is not required for the formation of first wave endocrine cells in mice [11,12], but promotes second wave islet cell formation [13,14]. *Hb9 *is expressed in a pattern overlapping with *Pdx1 *in the early progenitors of the dorsal and ventral buds [15]. After formation of pancreatic buds, *Hb9 *is initially downregulated, and is activated again in differentiating beta cells [16,17]. Loss of function *Hb9*^-/- ^mice lack the dorsal bud, while ventral bud-derived islets are smaller and have decreased numbers of incompletely differentiated beta cells [16,17].

In zebrafish, morpholino knockdown approaches have been used to study pancreatic function of *hb9 *and *pdx1 *[18-20]. Following morpholino-mediated *pdx1 *knockdown, embryos showed delayed appearance of both exocrine and endocrine cells but displayed an almost normal overall structure of the pancreas by 5 days post fertilization (dpf) [18,19]. This reported recovery raises questions about the requirement for *pdx1 *in progenitor specification in zebrafish. *hb9 *is expressed broadly in the endoderm during early somite stages and becomes restricted to insulin-producing beta cells after onset of endocrine differentiation at 15 hpf. Morpholino knockdown analysis revealed a requirement for *hb9 *in early beta cells [20]. The coexpression of *pdx1 *and *hb9 *in beta cell progenitors suggests possible functional interactions that have as yet not been assessed.

Differentiation of beta cells from progenitors is coordinated with morphogenetic events that include delamination of precursors from the ductal epithelium, migration and islet assembly. Real-time imaging of this dynamic process has not been previously achieved, as direct observation is prevented by the deep interior location of the pancreas. Such studies are feasible in transparent zebrafish embryos. However, as visual access to the developing gut is obstructed by the large yolk cell, observation of endocrine cell emergence requires the implementation of new imaging approaches.

Regulation of cell fate decisions in pancreatic endocrine progenitors involves Notch-regulated lateral inhibition that in mouse leads to increased *Ngn3 *expression in a subset of cells within the nascent duct epithelium and induction of proendocrine transcription factors such as *NeuroD *[10,21,22]. Although the presumed zebrafish *ngn3 *ortholog appears not to be functionally equivalent to the mouse gene based on timing and localization of expression [6,23], *NeuroD *is expressed in early precursors that give rise to all endocrine cells in both mouse and zebrafish [24-27]. In mouse, NeuroD was found to have the capacity to drive endocrine differentiation in pancreatic progenitors [28]. NeuroD expression is maintained in insulin-positive beta cells, while it is downregulated in the other endocrine cell types [26].

In this study, we aimed to achieve a better understanding of beta cell progenitor specification and behavior, and the dependence of these processes on *pdx1 *and *hb9*. After early reduction of beta cell numbers following morpholino-mediated depletion of *hb9 *or *pdx1*, there is recovery of *insulin *expression at late stages. In contrast, loss of both *pdx1 *and *hb9 *leads to an essentially complete and persistent absence of beta cells. A detailed analysis of endocrine precursors in wild-type and morpholino-treated embryos using a *NeuroD:EGFP *transgenic line revealed cooperative activity of *hb9 *and *pdx1 *in establishing beta cell fate. Importantly, we define a requirement for *pdx1 *in latent, duct-associated, Notch-responsive progenitors responsible for the production of second wave endocrine cells. These investigations were facilitated by the establishment of new protocols for *in vivo *timelapse imaging that allow visualization of delaminating and migrating endocrine precursors at high resolution.

## Results

### Beta cell loss following simultaneous knockdown of *hb9 *and *pdx1*

**Figure 1:**
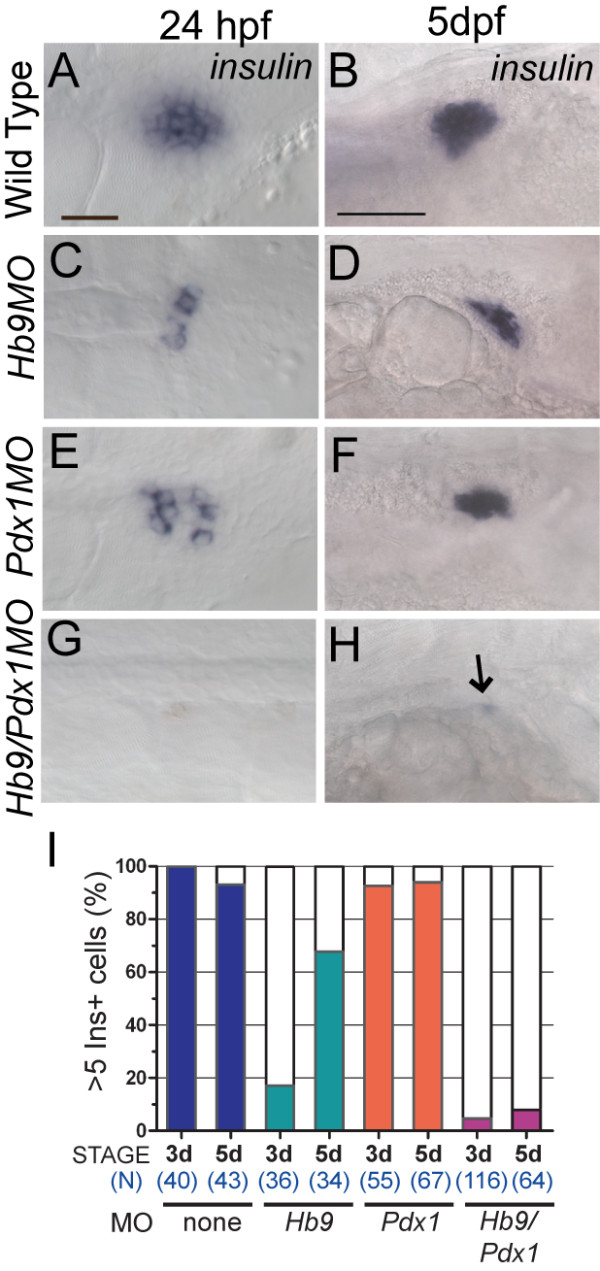
***ins *mRNA expression in *hb9 *and *pdx1 *morphants**. *In situ *detection of *ins *mRNA in wild-type embryos **(A,B)**, *hb9 *morphants **(C,D)**, *pdx1 *morphants **(E,F) **and *hb9*/*pdx1 *double morphants **(G,H) **at 24 h post fertilization (hpf) and 5 days post fertilization (dpf). In *hb9 *and *pdx1 *morphants the number of *ins *expressing cells is strongly reduced at 24 hpf as compared to control embryos (A,C,E) but has substantially increased by 5 dpf (D,F). (G,H) *ins *expression is missing in most double morphants (arrow in H marks a single *ins *positive cell). Embryos are shown from ventral (24 hpf) and lateral (5 dpf) view with anterior to the right. Scale bars correspond to 20 μm (24 hpf) and 50 μm (5 dpf). **(I) **Proportion of embryos showing > 5 Ins+ cells in control and morphant embryos at 3 dpf and 5 dpf. Beta cells emerge between 3 dpf and 5 dpf in *hb9 *and *pdx1 *morphants, while there is persistent absence in the *hb9/pdx1 *double morphant.

To determine how beta cell differentiation is affected by the depletion of *hb9 *and *pdx1*, we examined *insulin *(*ins*) expression over a time window of 5 days following morpholino (MO) injection. Consistent with previous studies [18,20,29], *hb9 *and *pdx1 *MO-treated embryos (morphants) analyzed by RNA *in situ *hybridization at 24 hpf had a marked decrease in *ins *expression (Figure 1C,E, compared to A). As seen in previous analyses of *pdx1 *morphants, we now show that also in *hb9 *morphants there was a substantial recovery of beta cell development and *ins *expression by 5 dpf (Figure 1D,F,I).

We next asked the question whether *hb9 *and *pdx1 *might cooperate in beta cell formation by coinjecting embryos with *hb9 *and *pdx1 *morpholinos. This resulted in an almost complete lack of *ins *expression at 24 hpf (Figure 1G). This phenotype persisted, and at 5 dpf and 9 dpf, *hb9/pdx1 *morphants showed virtually no *ins *expression (at 5 dpf 92% < 5 *ins *cells, n = 64; Figure 1H,I and at 9 dpf 93% < 5 *ins *cells, n = 14).

To exclude that pancreatic development is globally disrupted in the double morphant, we examined expression of the exocrine marker carboxypeptidase A (Cpa). As in the *pdx1 *morphants [18,19], *hb9/pdx1 *double morphants show substantial development of exocrine tissue at 4 dpf [see Additional file 1]. We also asked whether *pdx1 *expression returns at later stages in morpholino-injected embryos, by examining Pdx1 protein in morphants. At 28 hpf and 48 hpf, Pdx1 expression is entirely absent in the developing gut and pancreas of *pdx1 *morphants (n = 13 [see Additional file 2A-F]). At 84 hpf during normal development, Pdx1 is strongly expressed in cells of the islet with lower expression in the exocrine pancreas (n = 8 [see Additional file 2G]), while *pdx1 *morphants have low levels of Pdx1 protein in exocrine pancreas and islet at this stage (n = 8 [see Additional file 2H]). Thus, the morphant is not completely devoid of Pdx1 at late stages, but a low level of expression is observed that allows rescue of exocrine development and may account for the recovery of beta cells.

### Perturbed endocrine precursor formation in *hb9/pdx1 *knockdown embryos

Two mechanisms could account for the persistent absence of beta cells in the *hb9/pdx1 *double morphants: endocrine precursors may not be specified, or they may fail to differentiate. To distinguish these possibilities we analyzed green fluorescent protein (GFP) expression in morphant embryos transgenic for *TgBAC(NeuroD:EGFP)nl1 *[30]. These fish express enhanced green fluorescent protein (EGFP) integrated into the *NeuroD *locus contained in a bacterial artificial chromosome (BAC) by homologous recombination. EGFP expression has been reported to recapitulate *NeuroD *expression in the nervous system. We now confirm that EGFP in *TgBAC(NeuroD:EGFP)nl1 *embryos is first expressed in dispersed cells of the prepancreatic endoderm, which migrate posteriorly and cluster to form the principal islet by 24 hpf [see Additional files 3A and 4]. In a subset of cells there is coexpression of endocrine hormones [see Additional file 3B-G], recapitulating endogenous *NeuroD *expression in islet cell precursors [25,27]. In addition, the stability of EGFP allowed us to trace endocrine precursors during maturation after the downregulation of the endogenous *NeuroD*.

**Figure 2:**
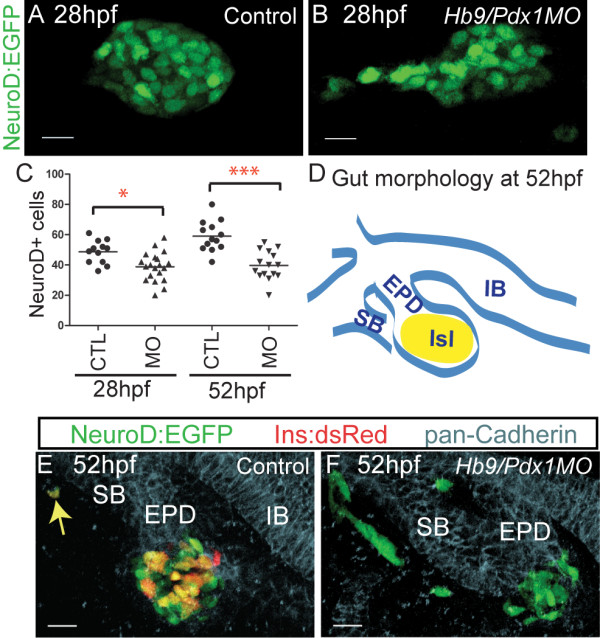
**Disrupted precursor specification and differentiation in *hb9/pdx1 *double morphants**. **(A,B) **Projections of confocal image stacks of native enhanced green fluorescent protein (EGFP) expression in control uninjected and in *hb9/pdx1 *morpholino injected *TgBAC(NeuroD:EGFP)nl1 *embryos at 28 h post fertilization (hpf). In morphants, the clustering of EGFP labeled cells is disrupted (B) and the number of EGFP expressing cells is reduced as compared to controls **(C)**. By 52 hpf, *NeuroD:EGFP *cell numbers have increased in control embryos, but not changed in the morphants ((C), *P < 0.05, ***P < 0.001 as determined by one-way analysis of variance (ANOVA) with Bonferroni's post test). **(D) **Schematic depicting overall gut morphology in 52 hpf embryos. EPD, extrapancreatic duct; IB, intestinal bulb; ISL, islet; SB, swim bladder. **(E,F) **Confocal image projections of control uninjected (E) and *hb9*/*pdx1 *morpholino injected (F) *TgBAC(NeuroD:EGFP)nl1*;*Tg(ins:dsRed)m1018 *embryos at 52 hpf that were costained with antibodies against pan-cadherin (light blue) to outline epithelial structures. In control embryos, EGFP+ and EGFP+/DsRed+ cells are mainly found in the islet. A rare EGFP+/DsRed+ cell is seen in a distal location (E, arrow). In double morphants, few to no EGFP/DsRed-expressing cells can be detected and many EGFP+ cells are located at ectopic positions outside the developing gut epithelium (F). Scale bar = 15 μM in all panels. All embryos are shown from ventral view, anterior to the left.

To assess endocrine precursor specification in *hb9/pdx1 *double morphants, we quantified pancreatic *EGFP+ *cells at 28 hpf, that is, before the emergence of the ventral bud. At 28 hpf, double morphant embryos displayed a slightly reduced number of EGFP positive cells as compared to controls (Figure 2A-C). In addition, while EGFP+ cells in control embryos formed a compact islet (n = 12), 50% of double morphants (n = 20) showed loosely clustered or even widely separated GFP+ cells (Figure 2B), suggesting disrupted migration. Thus, early endocrine cells were specified in these morphants, albeit at reduced levels, and the residual endocrine cells displayed defects in migration.

We next examined endocrine cell specification and differentiation at 52 hpf, after the fusion of dorsal and ventral buds. For this analysis we used embryos that were double transgenic for *TgBAC(NeuroD:EGFP)nl1 *and *Tg(ins:dsRed)m1018*, enabling simultaneous visualization of progenitors and differentiated beta cells by EGFP and dsRed, respectively, and in addition immunostained the embryos for pancadherin to delineate gut morphology (Figure 2D-F). In control embryos, the average number of EGFP+ cells increased by 20% between 28 hpf and 52 hpf (from 49 to 59 cells), but these remained essentially constant in number in the double morphants (average 39 cells; Figure 2C). Control embryos had an average of 18 EGFP+/DsRed+ cells (n = 12), compared to double morphants that had few to no DsRed+ cells (0 to 5 EGFP+/DsRed+ cells in 14/14 embryos) (Figure 2E,F). Furthermore, 47% (7/15) of double morphant embryos showed clusters of EGFP+/DsRed- cells located at a distance from the islet and outside of the pancadherin stained gut epithelium (Figure 2F). Only one ectopic EGFP+ cell was seen in a single control embryo (n = 12), and this cell also displayed robust DsRed expression (Figure 2E). As DsRed takes 24 h to reach its half maximal fluorescence intensity [31], this likely represents a rare displaced early rather than a late beta cell. In summary, our analyses show that early endocrine cell specification occurred in *hb9/pdx1 *double morphants, and therefore a deficit in differentiation contributes to the absence of *ins*-expressing cells at later developmental stages. In addition, the formation of second wave endocrine cells was disrupted, as the number of *NeuroD:EGFP *cells did not increase after 28 hpf in double morphants.

### Loss of late forming *NeuroD:EGFP *cells in *pdx1 *morphants

**Figure 3:**
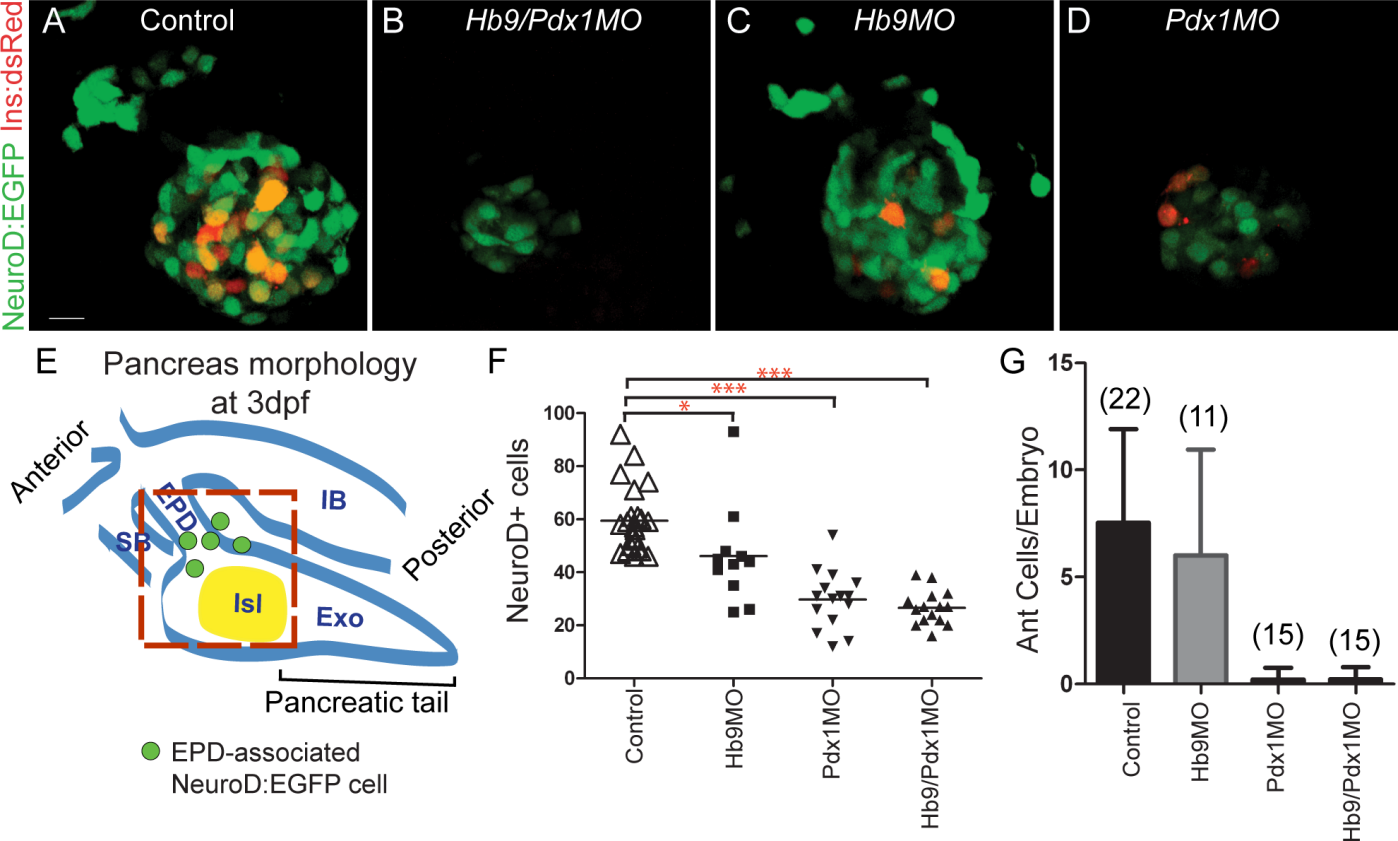
**Absence of ventral bud endocrine precursors in *hb9/pdx1 *and *pdx1 *morphant embryos**. **(A-D) **Projections of confocal stacks showing native fluorescence of 3 days post fertilization (dpf) *TgBAC(NeuroD:EGFP)nl1*; *Tg(ins:dsRed)m1018 *embryos. In uninjected control embryos (A) and in *hb9 *morphants (C), enhanced green fluorescent protein (EGFP)+ cells are found in the islet and in smaller numbers also anterior to the islet. In *hb9/pdx1 *double morphants (B) and *pdx1 *morphants (D), the number of islet-associated EGFP+ cells is strongly reduced and anterior cells are missing. Few *Ins:DsRed *cells are present in *pdx1 *single morphants at 3 dpf (D), reflecting slow maturation of the DsRed fluorophore. **(E) **Schematic of pancreas morphology at 3 dpf. The principal islet (Isl) at this stage is primarily dorsal bud derived. The ventral bud contributes new *NeuroD:EGFP *cells (green circles), and generates exocrine pancreas (Exo) and extrapancreatic duct (EPD). IB, intestinal bulb; SB, swim bladder. Red box delineates region included for quantitation of islet and newly emerging anterior endocrine cells. **(F) **Quantitation of pancreatic EGFP+ cells (*P < 0.05, ***P < 0.001 with *P *values determined using one-way analysis of variance (ANOVA) with Bonferroni's post test). **(G) **Quantitation of anteriorly positioned EGFP+ cells in uninjected and morpholino injected embryos (error bars indicate standard deviation from the mean). Scale bar = 10 μM.

We next analyzed *NeuroD:EGFP *cells in the islet of morphant embryos at 3 dpf to determine if emergence of second wave endocrine cells was delayed. Strikingly, *hb9/pdx1 *double morphants displayed a persistently low number of *NeuroD:EGFP*+ cells in the principal islet, which even appears reduced relative to earlier time points when only cells in the vicinity of islet and extrapancreatic duct were counted (Figure 3E), and distal cells outside of the gut were not included (Figure 2F).

As Pdx1 is required in second wave endocrine progenitors in mouse [13,14], we hypothesized that the defect in late endocrine cell formation in double morphants could be due to loss of *pdx1 *only. To assess the individual contributions of *pdx1 *and *hb9 *to late endocrine cell formation, we quantitated NeuroD cell number in single morphants. At 3 dpf, EGFP+ cells in *hb9 *morphants were only slightly reduced relative to controls, whereas in *pdx1 *morphants EGFP+ cells were reduced by 50%, similar to the number observed in double morphants (Figure 3A-D, F). In addition, control embryos had EGFP+ cells located anterior to the islet that were absent in double morphant embryos (Figure 3B). These cells occupied a position corresponding to that of previously described late-arising endocrine cells [4,32]. Strikingly, in *pdx1 *morphants but not in *hb9 *morphants, the anterior EGFP+ cells were also absent (Figure 3C,D,G). Thus, the formation of late endocrine cells specifically depends on *pdx1*, while early endocrine cells were specified in the absence of *pdx1 *or *hb9 *[see Additional file 5].

We also confirmed the persistent deficiency in new endocrine cell formation by examining *NeuroD:EGFP+ *cells in the principal islet at 5 dpf in *pdx1 *morphants as compared to control embryos, and found that *NeuroD:EGFP *cells remained dramatically reduced [see Additional file 6A]. Furthermore, in *pdx1 *morphants (n = 7), *NeuroD:EGFP+ *cells in the principal islet expressed Pdx1 at low levels, as compared to robust expression in controls [see Additional file 6C]. As *ins*-expressing cells have increased by 3 dpf in *pdx1 *morphants (Figure 1I), these beta cells originated from early specified, first wave endocrine precursors that differentiate under conditions of deficient Pdx1.

### Extrapancreatic duct-associated *NeuroD:EGFP *cells are migratory endocrine precursors

**Figure 4:**
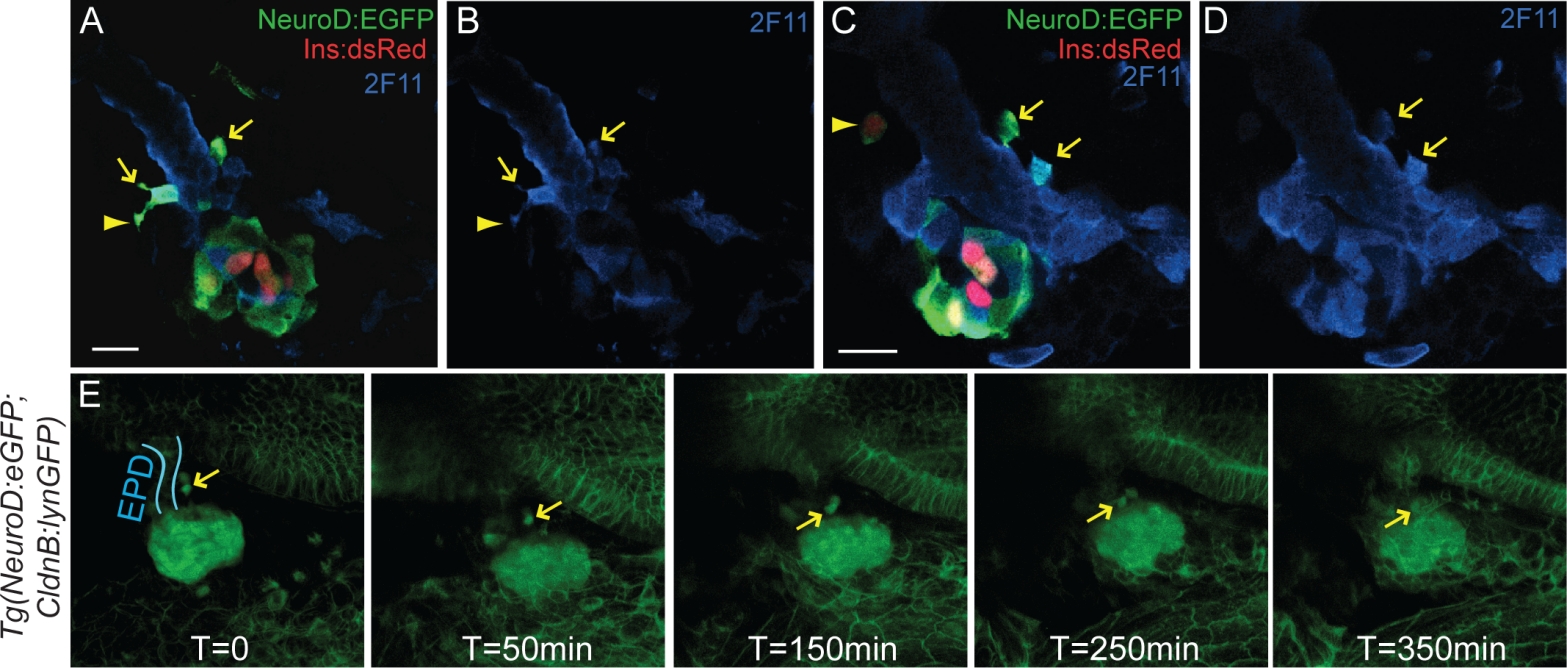
**Anterior *NeuroD:EGFP *cells are migratory endocrine precursors**. **(A-D) **Confocal sections of 3 days post fertilization (dpf) *TgBAC(NeuroD:EGFP)nl1*; *Tg(ins:dsRed)m1018 *embryos colabeled with antibodies against green fluorescent protein (GFP) and duct marker 2F11 (in blue). GFP+/2F11+ cells emerge from the duct (arrows), some with long projections typical of migrating cells (A, B, arrowhead). An individual cell that has separated from the duct is weakly DsRed positive (C, arrowhead). **(E) **Confocal image projections from a timelapse sequence of a 3 dpf *TgBAC(NeuroD:EGFP)nl1;Tg(-8.0cldnb:lynEGFP)zf106 *double transgenic embryo [see Additional file 8]. Membrane-linked enhanced green fluorescent protein (EGFP) in *Tg(-8.0cldnb:lynEGFP)zf106 *embryos delineates the gut epithelium (blue outline = extrapancreatic duct (EPD)) while robust cytoplasmic EGFP is present in NeuroD-expressing cells. Two *NeuroD:EGFP *cells (arrow) adjacent to the EPD migrate to the principal islet. All embryos are shown from ventral view, with the anterior to the left. Scale bar = 15 μM.

Second wave endocrine cells arise in association with the developing ductal epithelium [10,32]. To precisely localize the site at which *pdx1*-dependent anterior endocrine precursors arise, we immunostained *TgBAC(NeuroD:EGFP)nl1*;*Tg(ins:dsRed)m1018 *embryos at 3 dpf for the duct marker 2F11. NeuroD-expressing cells were found along the extrapancreatic duct (EPD), showed coexpression of 2F11, and even appeared to be emerging from the duct, with elongated processes characteristic of delaminating and migrating cells (Figure 4A-D). In support of their identity as endocrine precursors, occasionally such anterior *NeuroD:EGFP *cells expressed low levels of *Ins:DsRed *and thus correspond to newly differentiating beta cells (Figure 4C [see Additional file 7A]). This was confirmed by insulin (Ins) antibody staining [see Additional file 7C].

To document the emergence of these anterior endocrine cells, we developed a timelapse imaging technique based on a method described for imaging the central nervous system [33]. The large yolk cell, which normally obstructs the deeply located pancreas, was paralyzed and then extracted to establish a clear ventral view. This procedure did not interfere with morphogenetic processes. Using this novel method, we performed timelapse analysis of *TgBAC(NeuroD:EGFP)nl1*;*Tg(-8.0cldnb:lynEGFP)zf106 *embryos at 3 dpf. In double transgenic embryos, we recorded active delamination and clustering of *NeuroD:EGFP*-positive cells adjacent to the extrapancreatic duct and migration of cells towards the principal islet (Figure 4E [see Additional file 8]). Thus, our method allows the visualization of dynamic cell behaviors in the context of the developing organ in real time, and unambiguously demonstrates that new endocrine cells emerge from the duct epithelium and join the principal islet.

Having established that second wave endocrine precursors emerge from the duct, we analyzed if their absence in *pdx1 *morphants could be caused by a global duct defect. At 72 hpf, duct development appeared normal in morphant embryos, as demonstrated by a normal pattern of GFP-labeled duct cells in *Tg(-3.5nkx2.2a:GFP)ia3 *transgenics [34] [see Additional file 7D-G]. Furthermore, *Tg(-8.0cldnb:lynEGFP)zf106 *embryos, in which EGFP outlines membranes of the gut epithelium, revealed normal cellular structure of the extrapancreatic duct in *pdx1 *morphants as compared to controls [see Additional file 7H]. We examined Pdx1 expression in these embryos to address whether Pdx1 might have a role within the duct as new endocrine cells emerge. Pdx1 was highly expressed throughout the extrapancreatic duct and proximal pancreas in control embryos, while expression was very weak in *pdx1 *morphants [see Additional file 7J]. This analysis indicates that depletion of Pdx1 eliminated the ability of duct-associated cells to give rise to endocrine precursors and argues against an overall perturbation of the duct.

### Post-mitotic origin of late emerging endocrine cells

**Figure 5:**
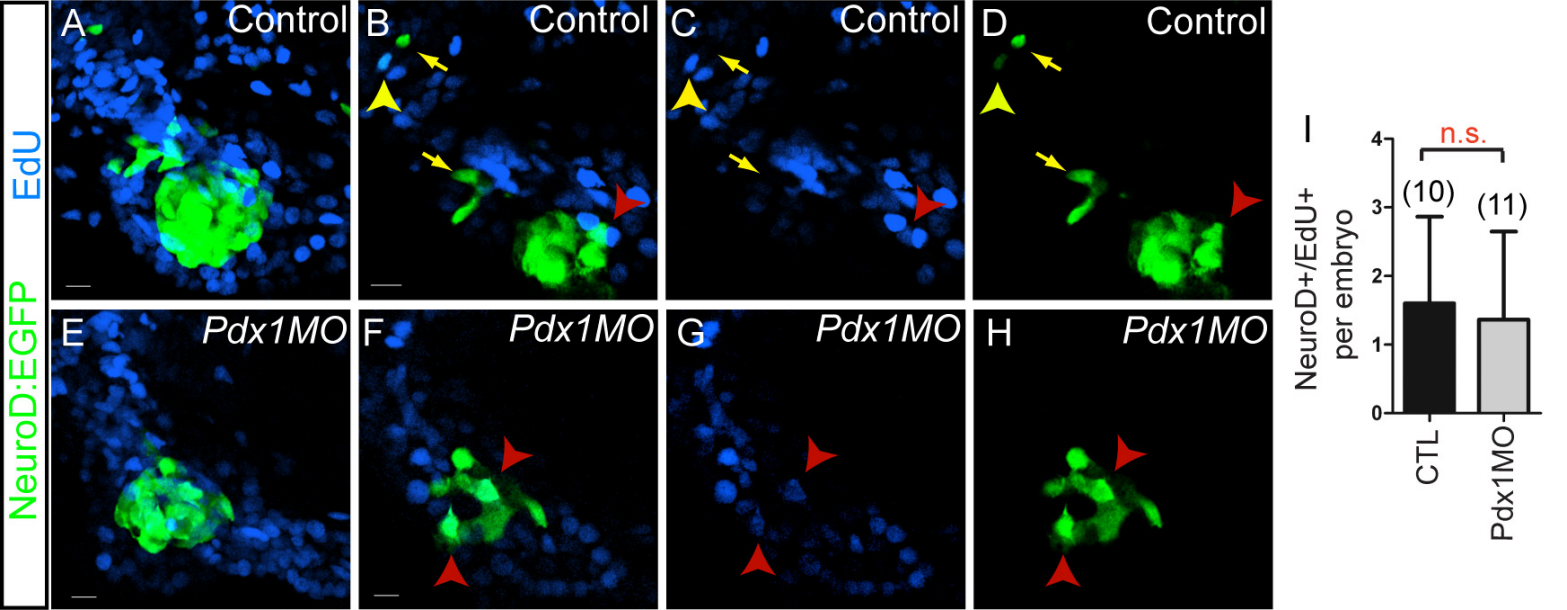
**Contribution of cell proliferation to late endocrine cell expansion**. *TgBAC(NeuroD:EGFP)nl1 *embryos incubated with EdU from 28 to 76 h post fertilization (hpf), followed by EdU detection and green fluorescent protein (GFP) antibody staining. **(A, E) **Confocal projections and single plane views **(B-D, F-H)**, showing pancreatic region of control (A-D), and *pdx1 *morphant (E-H) embryos. (B-D) In control embryos, EdU/NeuroD:enhanced green fluorescent protein (EGFP) colabeled cells are located at the periphery of the islet (red arrowhead) and in the region of the extrapancreatic duct (EPD) (yellow arrowhead). Most islet cells are EdU- (C compared to D) and additional EdU-/NeuroD:EGFP+ cells are found along the EPD (yellow arrows). (F-H) In *pdx1 *morphants, few EdU/NeuroD:EGFP colabeled cells are found within the islet (red arrowhead). **(I) **Quantitation of EdU/NeuroD:EGFP cells per embryo in wild-type and *pdx1 *morphant embryos, showing mean and standard deviation. Scale bar = 10 μm. NS, not significant; *P *> 0.05 as determined by unpaired t test.

We next asked the question whether late endocrine precursors arise from a mitotically active cell population. We tested this by incubating control and *pdx1 *morphant *TgBAC(NeuroD:EGFP)nl1 *embryos with nucleotide analog EdU from 28 hpf to 76 hpf. This extended incubation is expected to label dividing NeuroD+ endocrine precursors and dividing duct cells whose progeny differentiated into NeuroD-expressing endocrine cells. At 76 hpf, an average of 1.6 *NeuroD:EGFP *cells/embryo in control embryos were EdU positive, and their number in *pdx1 *morphants was not significantly different (1.4 NeuroD+/EdU+ cells/embryo (Figure 5I). In addition, rare EdU+/NeuroD+ cells were found at the periphery of the islet and distal to the islet, along the extrapancreatic duct in control embryos (Figure 5A-D). In summary, the NeuroD+/EdU+ double-labeled cells (average 1.6/embryo) cannot account for the appearance of ten additional NeuroD-expressing cells that emerge in embryos between 28 hpf and 76 hpf. Furthermore, most NeuroD+ cells outside of the principal islet were EdU-, indicating that late endocrine cells appearing after 28 hpf arose from post-mitotic precursors. The *pdx1 *morphant showed a similar low number of NeuroD+/EdU+ cells, which were all associated with the islet (Figure 5E-H). Our data therefore suggest that late endocrine cells emerge predominantly from a postmitotic cell type.

As proliferating cells were found directly adjacent to the *NeuroD:EGFP*+ islet, we next used the *Tg(ela3l:EGFP)gz2 *allele that labels exocrine pancreas [35] to define their identity. Incubation of embryos from 28 to 76 hpf with EdU, followed by a 12-h chase, resulted in extensive EdU labeling of the GFP+ exocrine pancreas in control as well as in *pdx1 *morphant embryos [see Additional file 9]. Therefore, ventral bud cells forming the exocrine pancreas are highly proliferative during this time period, and in *pdx1 *morphants no general proliferation defect exists in the pancreas. We conclude that beta cell recovery in *pdx1 *morphants is not accompanied by activated proliferation in progenitors or differentiated beta cells, but occurs through delayed precursor differentiation.

### Notch-responsive beta cell progenitors require *pdx1*

The zebrafish intrapancreatic duct is a ventral bud derivative containing Notch-responsive endocrine progenitors that begin to differentiate and assemble into secondary islets after 3 dpf [5,7]. Blocking Notch signaling after 3 dpf was previously shown to induce differentiation of endocrine cells from duct-associated progenitors [5,7]. We assessed whether the latent endocrine progenitors associated with the intrapancreatic duct also arise in a *pdx1*-dependent manner.

**Figure 6:**
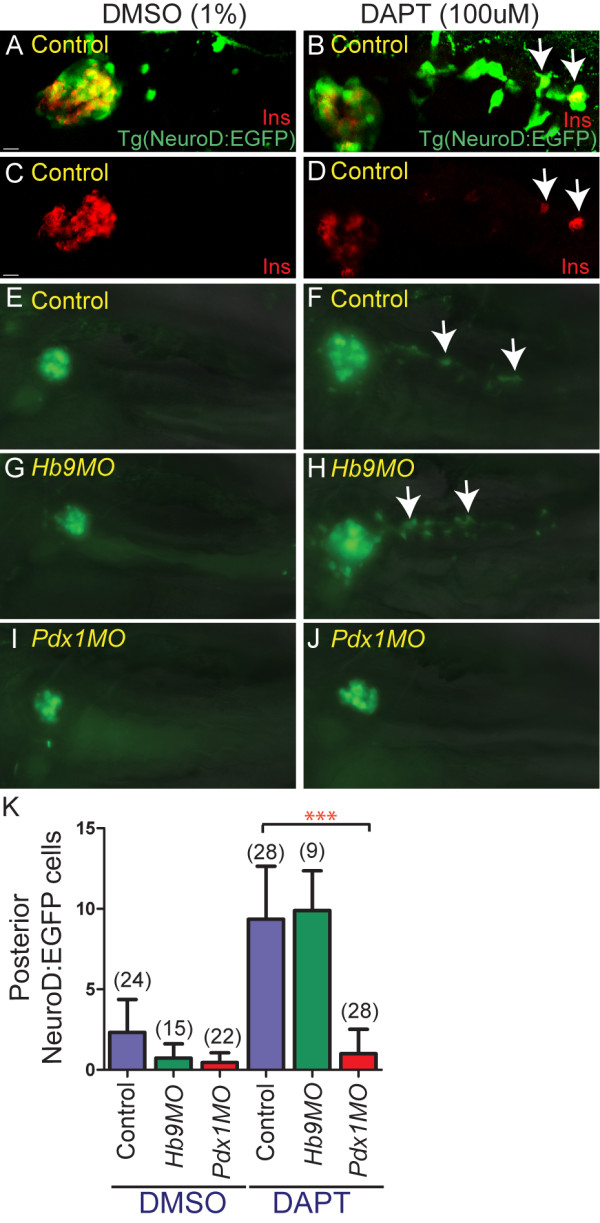
**Notch-responsive endocrine progenitors are absent in *pdx1 *morphants**. **(A-D) **Confocal image projections of *Tg(NeuroD:EGFP)nl1 *embryos immunostained for green fluorescent protein (GFP) and insulin. Optical sections that obscure the posterior pancreatic tail have not been included. **(E-J) **Native enhanced green fluorescent protein (EGFP) expression in the pancreas of living *TgBAC(NeuroD:EGFP)nl1 *embryos at 5.5 days post fertilization (dpf), with overlay of concurrently acquired brightfield image. Shown are uninjected control embryos (A-D, E, F), *hb9 *morphants (G,H) and *pdx1 *morphants (I,J) treated either with vehicle (dimethylsulfoxide (DMSO)) (A,C,E,G,I) or 100 mM *N*-[*N*-(3,5-difluorophenacetyl)-L-alanyl]-*S*-phenylglycine *t*-butyl ester (DAPT) (B,D,F,H,J) from 3 to 5 dpf. DAPT treatment in control embryos leads to the induction of EGFP expression in the pancreatic tail (A,B,E,F) and of Ins in individual of these EGFP+ cells (B, D). In *hb9 *morphants the patterns of EGFP expression in DMSO-treated and DAPT-treated embryos are similar to that in control embryos (E-H), while *pdx1 *morphants show decreased EGFP+ cells in the principal islet and virtually no posterior EGFP+ cells are induced by DAPT (I,J). All embryos lateral view, anterior to the left. Scale bar = 10 μM. **(K) **Quantitation of *NeuroD:EGFP *cells induced in the pancreatic tail following DAPT treatment in control and morphant embryos. Graphed are the mean and SD for (n) number of embryos. *P *values were determined using one-way analysis of variance (ANOVA) with Bonferroni's post test, ***P < 0.001.

To visualize the emergence of endocrine cells from the intrapancreatic duct, we first used a Notch inhibitor (*N*-[*N*-(3,5-difluorophenacetyl)-L-alanyl]-*S*-phenylglycine *t*-butyl ester (DAPT)) to induce endocrine differentiation and *NeuroD:EGFP *expression in latent progenitors. In control experiments, treatment of *TgBAC(NeuroD:EGFP)nl1 *embryos with DAPT from 3 to 5 dpf caused an increase of cells in the principal islet and a fourfold increase of GFP labeled cells within the pancreatic tail as compared to vehicle-treated larvae (Figure 6A,E,F,K). Overlapping expression of Ins and GFP following DAPT treatment confirmed that newly-induced *NeuroD:EGFP *cells in the pancreatic tail can differentiate into beta cells (Figure 6B,D).

**Figure 7:**
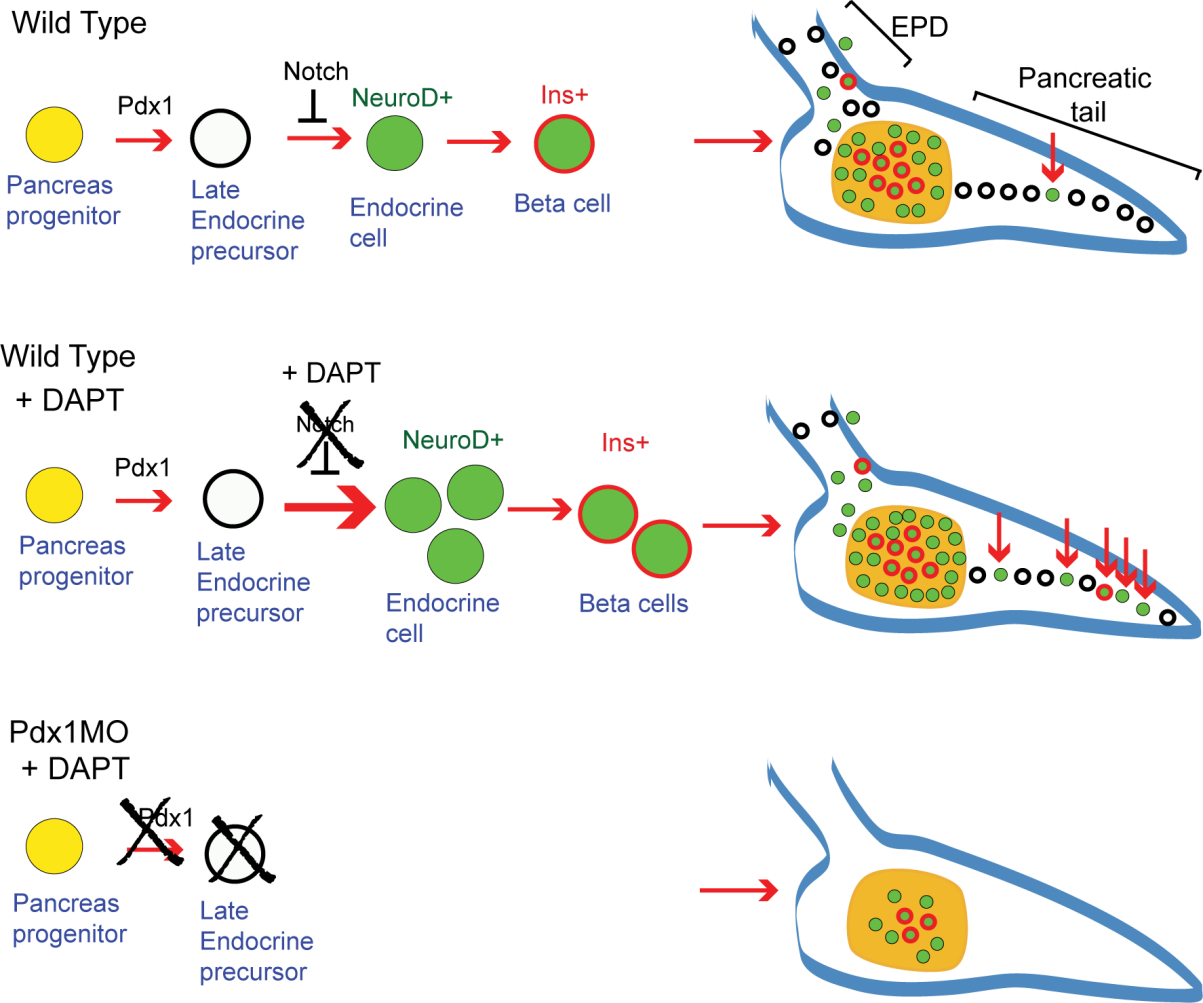
**Schematic of Notch-responsive progenitors**. In wild-type embryos (top), an early pancreatic progenitor (yellow) gives rise to early endocrine cells (green) as well as progenitors for late endocrine cells (white) located in the extrapancreatic duct (EPD) and pancreatic tail. Notch signaling maintains these latent progenitors in an undifferentiated state. Inhibition of Notch signaling by treatment with *N*-[*N*-(3,5-difluorophenacetyl)-L-alanyl]-*S*-phenylglycine *t*-butyl ester (DAPT) (center) activates differentiation of the latent progenitors, leading to new endocrine cell formation in the principal islet and pancreatic tail (red arrows). In *pdx1 *morphants (bottom), early endocrine specification occurs but Notch-responsive progenitors for late endocrine cells are absent or hindered in differentiation, which can be demonstrated by treatment with DAPT.

We next analyzed if these Notch-responsive cells were present in *pdx1 *and *hb9 *morphants. We found virtually no *NeuroD:EGFP *induction following DAPT treatment in *pdx1 *morphants, while DAPT treatment in *hb9 *morphants resulted in an increased number of *NeuroD:EGFP+ *cells similar to controls (Figure 6G-K). Immunostaining for duct marker 2F11 indicated that intrapancreatic duct formation in *pdx1 *morphants was comparable to controls [see Additional file 10A]. Overall, this indicates that Notch-responsive progenitors for late-forming endocrine cells, that contribute new cells to both principal islet expansion and secondary islet formation, require *pdx1 *(Figure 7).

## Discussion

In this work, we determined that both *hb9 *and *pdx1 *have roles in early beta cell formation and that *pdx1 *is distinctly required for second wave endocrine progenitor specification, which generates endocrine cells that expand the principal islet and form secondary islets. Prior studies of *pdx1 *morphant zebrafish did not quantitatively assess endocrine cell dynamics, and therefore the requirement for Pdx1 in formation of progenitors for late endocrine cells went unrecognized. Our new imaging approach allowed us to visualize the behavior of emerging endocrine cells in real time and define the source of the late endocrine progenitor cells. Specifically, we show that they originate and delaminate from the ductal epithelium in a process that resembles formation of second wave endocrine cells in mammals. Our findings identify a genetic requirement for formation of late endocrine cells, establish the site where the latent postmitotic cell type that gives rise to the endocrine progenitor resides, and describe a novel method to visualize morphogenetic events that occur during islet formation.

### Sources of beta cell recovery

Embryos deficient in either *pdx1 *or *hb9 *alone display an initial decrease in *ins *expression, followed by restoration of some *ins*-expressing cells by 5 dpf. We show here that in double morphants such a recovery does not occur, and define *pdx1 *and *hb9 *dependent steps in the generation and differentiation of two distinct endocrine cell populations arising in the early and late embryo. The early appearance of *NeuroD:EGFP*-expressing islet cells demonstrated that committed, first wave endocrine cells of the dorsal bud were specified despite the absence of *hb9 *and/or *pdx1*. By contrast, second wave endocrine cells strictly depend on *pdx1*. The apparent recovery of beta cells in *pdx1 *morphants thus results from delayed differentiation of early arising, dorsal bud-derived endocrine precursors in conditions of severely reduced Pdx1. Since we have detected *hb9 *in ventral bud-derived beta cells (D Mayer, RA Kimmel, unpublished results), and *Hb9 *knockout causes global beta cell deficits in mouse, delayed beta cell differentiation in *hb9 *morphants can be explained by restoration of protein function due to dilution of morpholino at the time when late endocrine cells emerge.

### Progenitors for late endocrine cell expansion

During organ development, progenitors are often set aside during the early stages to undergo later expansion and differentiation. In prior studies of beta cell proliferation during zebrafish embryogenesis using nucleotide analogs or proliferating cell nuclear antigen (PCNA) antibody, dividing beta cells were detected only very rarely or not at all [3,6,18,36,37]. To analyze and define the proliferative status of cells generating the late-arising endocrine cells, we used a protocol in which embryos were exposed to a long pulse of EdU covering the time window when late endocrine cells start emerging (from 28 to 76 hpf). This captures all cell divisions and also labels cells that proliferate prior to endocrine differentiation. Indeed, due to the prolonged labeling time, we observed a higher proportion of cells that had undergone proliferation as compared to previous studies, indicating that dividing progenitors that gave rise to beta cells as well as the rare beta cell mitoses were revealed. Nevertheless, the low number of EdU+ endocrine precursors and beta cells that we detected in normal development shows that the vast majority of late arising endocrine cells (appearing between 28 hpf and 76 hpf) have not undergone mitosis during the labeling period. Therefore, these cells differentiate directly from a precursor with low proliferative activity. Similarly, in the *pdx1 *morphant, proliferating beta cells or mitotic progenitors contribute only a minor fraction to the beta cells emerging after 28 hpf.

These studies indicate that, during normal development, the cell type that gives rise to late endocrine cells represents a subtype of ventral bud cells that is already set aside at 28 hpf, and that slowly divides in contrast to the rapidly dividing cells that form the exocrine pancreas. Previous analysis showed that the ventral pancreatic bud contains *ptf1a*-expressing cells that form the exocrine pancreas and a distinct population that responds to Notch and gives rise to endocrine and duct cells [5]. Our data indicates that within the non-exocrine cells, there is a population of non-proliferative cells that comprises latent endocrine progenitors.

### Function of Pdx1 in late endocrine cells

A major finding of this work is the requirement for *pdx1 *in specification of all late forming endocrine cells. Expression of *Pdx1 *is very dynamic in the developing pancreas and genetic analyses using conditional and hypomorphic *Pdx1 *mutant alleles in mouse have already indicated that Pdx1 is important in various specification and differentiation events [38-41]. In zebrafish, fate-mapping studies indicated that a spatial separation of the future pancreatic DB versus VB progenitors is already established shortly after gastrulation (10 hpf) [42,43]. Medium levels of *pdx1 *expression during early somitogenesis (10 to 15 hpf) correlate with VB fate specification [44]. Thus, already these early differences in *pdx1 *expression could provide permissive or instructive cues for the specification of latent progenitors that later give rise to second wave endocrine cells.

The developing extrapancreatic duct and the proximal pancreas express high levels of Pdx1 from 48 hpf ([32] and this study). In *pdx1 *morpholino-treated embryos, Pdx1 is diminished at least until 3 dpf, but overall formation of the extrapancreatic duct is not affected. An absence of duct-associated progenitors for second wave endocrine cells could result from duct cells being committed to a non-endocrine fate due to an early deficiency in Pdx1. Alternatively, deficient Pdx1 in duct cells might impede the generation of endocrine precursors from a latent progenitor. Several lines of research suggests that pancreatic ductal epithelium is heterogeneous and contains a limited numbers of cells that have the potential for endocrine differentiation [45]. We have here defined a strategy that eliminates endocrine cell formation without affecting duct morphology, which will be useful to help further characterize the latent progenitor cell type.

### Morphogenesis of late endocrine cells

Our transgenic and timelapse analyses provide new insights into the behavior of late-arising endocrine cells. *NeuroD:EGFP*-positive cells associated with the extrapancreatic duct defined by 2F11 antibody staining exhibited protrusions characteristic of delaminating cells. We developed a method for maintaining embryo viability after yolk removal, which allowed for the first time a clear visualization of developing late endocrine cells in living embryos. We delineated pancreatic progenitors in the context of the pancreatic duct and digestive system, and recorded migration of *NeuroD:EGFP *positive endocrine progenitors as they emerged from the duct and moved to the principal islet. This method provides the basis for future analysis of molecular regulators of these complex morphogenetic events during pancreatic islet development.

## Conclusions

These studies establish that *pdx1 *is essential for generating an endocrine cell progenitor population associated with the pancreatic duct in zebrafish. We show that formation of latent progenitors in the extrapancreatic and intrapancreatic duct requires *pdx1*, and provide evidence that they are slowly dividing cells, and are thus distinct from rapidly proliferating cells that form the exocrine tissue. Further, these latent progenitors differentiate in response to Notch inhibition. Our findings open up new approaches for examination of the specification and behavior of endocrine progenitors during zebrafish embryogenesis, in which these processes can now be visualized, and where large-scale chemical and pharmacologic screening can be applied. This can in turn provide clues as to how developmental programs can be harnessed and reactivated to generate replacement beta cells for more efficient treatment of diabetes.

## Methods

### Zebrafish transgenic lines

The following transgenic lines were used: *Tg(ins:dsRed)m1018 *(generous gift of Wolfgang Driever, University of Freiburg, Germany), *Tg(ela3l:EGFP)gz2 *[35]; *TgBAC(NeuroD:EGFP)nl1 *[30]; *Tg(-3.5nkx2.2a:GFP)ia3 *[34]; *Tg(-8.0cldnb:lynEGFP)zf106 *[46]. Zebrafish (*Danio rerio*) were maintained according to standard protocols.

### Morpholino injection

The following previously validated morpholinos, targeted against the initiation codon of *pdx1 *and *hb9*, respectively, were obtained from Gene Tools (Philomath, OR, USA): *pdx1*MO: 5'-GATAGTAATGCTCTTCCCGATTCAT-3' [18,19]; *hb9*MO: 5'-TTTTTAGATTTCTCCATCTGGCCCA-3' [20].

Each morpholino was prepared at a concentration of 1.5 mM. This solution was diluted to 750 μM in water and 2 to 8 ng of morpholino in a volume of approximately 1.5 nl was injected into one-cell stage embryos. In generating double morphants, the concentration of individual morpholinos was halved to keep the total amount of morpholino injected the same as for single morphants.

### *In situ *hybridization

Whole mount *in situ *hybridization was performed as described [47]. Digoxigenin antisense probes (Roche, Vienna, Austria) were synthesized for *insulin *[48]. As precise cell counting in older embryos becomes difficult with increasing cell numbers in a tight cluster, embryos with cell count > 5 were grouped together for quantitation.

### Antibody staining

Embryos were fixed in 4% paraformaldehyde (PFA) at 4°C (2 h to overnight, depending on stage) followed by proteinase K (Carl Roth, Karlsruhe, Germany) digestion (10 μg/ml in phosphate-buffered saline (PBS)), post fixation in 4% PFA, and incubation in blocking buffer containing 1% bovine serum albumin (BSA, AppliChem, Darmstadt, Germany), 2% Sheep serum (Sigma, Vienna, Austria), 1% Triton, and 1% dimethylsulfoxide (DMSO). Primary antibody was diluted in blocking buffer followed by overnight incubation at 4°C. Embryos were washed in PBS/BSA/Triton, and incubated overnight at 4°C in secondary antibody diluted 1:1,000 in blocking buffer, followed by extensive washing in PBST (PBS + 0.1% Tween 20). Primary antisera and dilutions were: guinea pig anti-insulin (1:200, Dako, Vienna, Austria), rabbit anti-glucagon (1:200, Dako), rabbit anti-pan-cadherin (1:500, Sigma), mouse anti-2F11 (1:100, generous gift from Julian Lewis, London Research Institute, UK), rabbit anti-somatostatin (1:200, Dako), rabbit anti-carboxypeptidase A (1:200, Chemicon, Vienna, Austria), mouse anti-GFP (1:200, Roche), rabbit anti-GFP (1:200, Torrey Pines Biolabs, East Orange, NJ, USA), guinea pig polyclonal anti-Pdx1 (1:200, generous gift from Chris Wright, Vanderbilt University, TN, USA). Alexa-conjugated secondary antibodies (1:1,000 dilution) were from Invitrogen (Lofer, Austria).

### Cell labeling with EdU

EdU staining was performed as previously described [37]. In brief, reagents for the Click-iT EdU Alexa Fluor 647 Assay (Invitrogen) were prepared according to manufacturer's protocol. Embryos were incubated in 0.5 mM EdU/0.4% DMSO in egg water (0.3 g/L Red Sea Coral Pro Salt in reverse osmosis H_2_O) followed by fixation in 4% PFA. Embryos were then washed in PBST, dehydrated with a methanol wash, and incubated in methanol at -20°C for 2 h. For the detection assay, larvae were rehydrated through a methanol series into PBST and manually deyolked. The larvae were treated with proteinase K (10 μg/ml) for 45 min, refixed in 4% PFA for 20 min, washed in PBST, and permeabilized in 1% DMSO/0.5% Triton/PBS. Incubation in Click-iT reaction cocktail was performed for 2 h at room temperature followed by rinses in PBST. Following the EdU detection reaction, larvae were incubated in blocking buffer and immunostaining was performed as described above.

### Microscopy

*Tg(ins:dsRed)m1018 *and *TgBAC(NeuroD:EGFP)nl1 *embryos for direct fluorescence imaging were fixed in 4% PFA at room temperature for 1 to 2 h (48 hpf and younger) or 2 to 3 h (older than 48 hpf), rinsed three times with PBST followed by manual yolk removal. Transgene and antibody immunostaining were imaged with a Zeiss LSM5 Exciter (Carl Zeiss, Vienna, Austria) confocal laser microscope, using a 40 × water immersion objective. Stacks of optical sections were recorded with a z-step ranging from 1 to 2 μm. Larvae for live imaging were anesthetized using 1 × Tricaine mesylate (0.003%, Sigma), immobilized in low melt agarose (1.5%, Biozym, Vienna, Austria) and imaged on a Leica DM6000B microscope (Leica Microsystems, Vienna, Austria) equipped with a SPOT-RT3 digital camera (Diagnostic Instruments, Inc., Sterling Heights, MI, USA), using a 20 × water objective. Fluorescence stack and differential interference contrast (DIC) images were captured using Visiview software (Visitron Systems, Puchheim, Germany). Fluorescence brightfield composite images were prepared with ImageJ (http://rsbweb.nih.gov/ij).

### Confocal timelapse analyses

Imaging of dorsal bud cell migration at 19 hpf was performed as previously described [37]. For timelapse imaging of older embryos, 3 dpf embryos were anesthetized with 1 × Tricaine mesylate diluted in egg water, then the yolk cell was paralyzed by injection of 40 mM AMP-PNP (adenosine 5' (β,γ-imido)triphosphate, Sigma) and gently extruded using a tungsten needle followed by separation of ventral membranes to expose digestive tract and pancreas. Embryos were mounted dorsal side down in 1.5% low-melt agarose and covered with L-15 medium (Invitrogen) diluted to 67% with sterile water, and supplemented with penicillin/streptomycin cocktail (1 × final, Invitrogen), glucose (10 mM final, AppliChem) and 1 × Tricaine mesylate. Embryos were imaged for 5 to 10 h in a temperature-controlled room at 28°C. Images were collected every 5 min, a total z-stack of 60 to 70 μm was acquired with a slice interval of 2.5 to 3.5 μM. Images were captured using a 40 × water objective on a Zeiss LSM Exciter5 equipped with Zen 2008 software (Carl Zeiss), followed by processing and assembly of still images and videos using LSM Image Browser (Carl Zeiss) and ImageJ.

### Quantitative image analysis

NeuroD cell numbers in 24 hpf to 3 dpf embryos were quantitated using Imaris 7.1.1 software (Bitplane, Zurich, Switzerland). The spot detection algorithm was applied to three-dimensional reconstructions of samples to identify cells using a spot size of 3 to 4 μm. Spots were filtered, confirmed and edited by visual inspection, and cell counts transferred to Prism (GraphPad Software, La Jolla, CA, USA) for statistical analyses. For proliferation analyses, spot detection was similarly performed for EdU+ and NeuroD+ cells, double labeling of cells was determined using the 'colocalize spots' function of Imaris with a threshold value of 3 μm.

### DAPT treatment

DAPT (Sigma) was prepared as a 10 mM stock solution in DMSO. Larvae were incubated in DAPT diluted to 100 μM in egg water starting at 3 dpf for 48 h. The DAPT solution was replaced after 24 h. The embryos were then rinsed in egg water and incubated an additional 12 h. Control embryos were incubated in 1% DMSO in egg water.

## Competing interests

The authors declare that they have no competing interests.

## Authors' contributions

RAK and DM designed the research. EE, RAK, LO, AW and DM performed research and analyzed data. RAK and DM wrote the paper. All authors read and approved the final manuscript.

## Supplementary Material

Additional file 1 **Exocrine pancreas development in *hb9/pdx1 *double morphants**. Confocal image projection of the pancreas of 4 days post fertilization (dpf) embryos immunostained for carboxypeptidase A (Cpa) and insulin (Ins). *hb9/pdx1 *double morphant embryos **(B) **with severely reduced Ins (insets, **(A)**, compared to B), show Cpa expression comparable to control embryos (A). All are in ventrolateral view. Scale bar = 30 μM. Click here for file

Additional file 2 **Pdx1 protein expression in *pdx1 *morphants**. Confocal projections of control and *pdx1 *morpholino-injected embryos at 28 h post fertilization (hpf) **(A-C) **and 48 hpf **(D-F)**, immunostained for Pdx1. (A-C) Control embryos express Pdx1 in the developing gut and pancreas. (D-F) *pdx1 *morphant embryos have no detectable Pdx1 expression. (B,E) Overlay of bright field with confocal projection of Pdx1 antibody staining, (C,F) single channel showing Pdx1 antibody. Dashed circle (E) indicates islet as determined from bright field images. Non-specific labeling by this antibody of somites lateral to the gut (asterisks) has been previously described [49]. Ventral view. Scale bar = 10 μM. Confocal projections **(G,H) **and single plane views **(I-L) **of control and *pdx1 *morphants at 84 hpf, immunostained for Pdx1 and carboxypeptidase A (Cpa) to indicate the exocrine pancreas. Control embryos show robust Pdx1 expression in the islet and weaker expression in the exocrine pancreas (G,I,K). *pdx1 *morphant embryos have low-level Pdx1 expression in the islet and exocrine pancreas (H,J,L). Lateral view, anterior to left. Scale bar = 30 μM. Click here for file

Additional file 3 **NeuroD: enhanced green fluorescent protein (EGFP) cells are endocrine precursors**. **(A) ***TgBAC(NeuroD:EGFP)nl1 *embryos, mounted dorsal side up, imaged by confocal timelapse microscopy starting at 19 h post fertilization (hpf). Images were captured every 15 min. Asterisks indicate fixed points of the embryo determined from the bright field image. Anterior is to the left. **(B-G) **Confocal image projection of 24 hpf *TgBAC(NeuroD:EGFP)nl1 *embryo immunostained for green fluorescent protein (GFP) and insulin (Ins) (B-D) and GFP and somatostatin (Sst) (E-G), showing overlap of NeuroD:EGFP expression with islet hormones in a subset of cells. All are ventral view. Scale bar = 15 μM. Click here for file

Additional file 4 **Migration of early endocrine cells**. Confocal timelapse imaging of *TgBAC(NeuroD:EGFP)nl1 *embryo beginning at 19 h post fertilization (hpf). Confocal stacks were collected every 15 min. Dorsal view, anterior to the left. Still images from this file were used in Additional file 3A. Click here for file

Additional file 5 **Additional table 1**. NeuroD: enhanced green fluorescent protein (EGFP) cells at 28 h post fertilization (hpf) in control, *hb9*- and *pdx1*- morphants. Click here for file

Additional file 6 **Larval NeuroD:EGFP and Pdx1 expression**. Confocal projections **(A,D) **and single plane views **(B,C,E,F) **of control (A-C) and *pdx1 *morpholino injected (D-F) *TgBAC(NeuroD:EGFP)nl1 *embryos at 5 days post fertilization (dpf), immunostained for Pdx1 and green fluorescent protein (GFP). (A-C) In control embryos, robust Pdx1 expression in the islet overlaps with GFP. (D-F) *pdx1 *morphant embryos have reduced Pdx1 expression and GFP+ cells in the islet as compared to control. Lateral view, anterior to left. Scale bar = 10 μM. Click here for file

Additional file 7 **Beta cell differentiation and duct development**. **(A,B) **Single plane, ventral view of *TgBAC(NeuroD:EGFP)nl1;Tg(ins:dsRed)m1018 *control embryo at 72 h post fertilization (hpf). With the red channel overexposed, anterior enhanced green fluorescent protein (EGFP)+ cells with low levels of InsDsRed can be detected (arrow). **(C) **Confocal projection of *TgBAC(NeuroD:EGFP)nl1 *embryo at 72 hpf immunostained for green fluorescent protein (GFP) and insulin (Ins). The Ins signal is overexposed to show low-expressing cells anterior to the principal islet. Scale bar = 10 μM. **(D-G) **Confocal projection of 84 hpf *Tg(-3.5nkx2.2a:GFP)ia3 *embryos immunostained for GFP and Ins. GFP expression delineates developing duct in control (D,F), and *pdx1 *morphant (E,G) embryos. EPD, extrapancreatic duct; IPD, intrapancreatic duct. Ventral view. Scale bar = 15 μM. **(H-K) **Single plane views of 72 hpf *Tg(-8.0cldnb:lynEGFP)zf106 *embryos immunostained for Pdx1 and GFP. Control embryos show robust Pdx1 expression in the proximal pancreas and EPD (H,J), as compared to weak expression in *pdx1 *morphants (I,K). (J) and (K) represent the red channel only from (H) and (I). Ventral view, anterior to left. Scale bar = 10 μM. Click here for file

Additional file 8 **NeuroD:enhanced green fluorescent protein (EGFP) cells migrate from duct to islet**. Timelapse confocal series of a 3 days post fertilization (dpf) *TgBAC(NeuroD:EGFP)nl1;Tg(-8.0cldnb:lynEGFP)zf106 *embryo imaged for 6 h from a ventral view with yolk removed. Images were collected every 5 min. Migrating cells are indicated by arrows in the initial frames. Still images from this file were used in Figure 4E. Click here for file

Additional file 9 **Proliferation during exocrine pancreas formation**. *Tg(ela3l:EGFP)gz2 *[35] embryos labeled with EdU from 24 to 72 h post fertilization (hpf), and fixed at 84 hpf. EdU detection (blue) was followed by antibody staining to label Elastase (Ela)-expressing exocrine cells (Ela:green fluorescent protein (GFP), green) and beta cells (insulin (Ins), red). **(A,C) **Three-dimensional confocal projections showing composite of Ela:GFP, EdU and Ins in control (A), and *pdx1 *morphant embryos (C) at 84 hpf. EdU labels exocrine pancreas extensively in control and morphants. **(B,D) **Single plane views of embryos as in (A) and (C), showing rare cells with EdU/Ins colabeling (arrow). For clarity, the GFP channel is not shown. **(E) **Quantitation of EdU/Ins colabeled cells per embryo in control and *pdx1 *morphant embryos, showing mean and standard deviation. NS, not significant; *P *> 0.05 as determined by unpaired t test. Anterior is to the left. Scale bar = 15 μm. Click here for file

Additional file 10 **Larval duct morphology**. Confocal projection of 6 days post fertilization (dpf) *TgBAC(NeuroD:EGFP)nl1 *embryos immunostained for green fluorescent protein (GFP) and 2F11. In control embryos **(A)**, 2F11 positive cells surround GFP+ islet cells and extend into the pancreatic tail. Single NeuroD+ cells can be found in the pancreas tail (arrow). **(B) ***pdx1 *morphant embryos, with fewer GFP+ cells in the islet, have similar 2F11 expression around the islet and in the pancreatic tail (arrows). Lateral view. Scale bar = 30 μM. Click here for file

## References

[B1] Guney MA Gannon M Pancreas cell fate Birth Defects Res C Embryo Today 2009 87 232 248 10.1002/bdrc.20156 19750517 PMC2755625

[B2] Solar M Cardalda C Houbracken I Martin M Maestro MA De Medts N Xu X Grau V Heimberg H Bouwens L Ferrer J Pancreatic exocrine duct cells give rise to insulin-producing beta cells during embryogenesis but not after birth Dev Cell 2009 17 849 860 10.1016/j.devcel.2009.11.003 20059954

[B3] Hesselson D Anderson RM Beinat M Stainier DY Distinct populations of quiescent and proliferative pancreatic beta-cells identified by HOTcre mediated labeling Proc Natl Acad Sci USA 2009 106 14896 14901 10.1073/pnas.0906348106 19706417 PMC2736433

[B4] Field HA Dong PD Beis D Stainier DY Formation of the digestive system in zebrafish. II. Pancreas morphogenesis Dev Biol 2003 261 197 208 10.1016/S0012-1606(03)00308-7 12941629

[B5] Wang Y Rovira M Yusuff S Parsons MJ Genetic inducible fate mapping in larval zebrafish reveals origins of adult insulin-producing {beta}-cells Development 2011 138 609 617 10.1242/dev.059097 21208992 PMC3026409

[B6] Moro E Gnugge L Braghetta P Bortolussi M Argenton F Analysis of beta cell proliferation dynamics in zebrafish Dev Biol 2009 332 299 308 10.1016/j.ydbio.2009.05.576 19500567

[B7] Parsons MJ Pisharath H Yusuff S Moore JC Siekmann AF Lawson N Leach SD Notch-responsive cells initiate the secondary transition in larval zebrafish pancreas Mech Dev 2009 126 898 912 10.1016/j.mod.2009.07.002 19595765 PMC3640481

[B8] Chen S Li C Yuan G Xie F Anatomical and histological observation on the pancreas in adult zebrafish Pancreas 2007 34 120 125 10.1097/01.mpa.0000246661.23128.8c 17198193

[B9] Bonal C Herrera PL Genes controlling pancreas ontogeny Int J Dev Biol 2008 52 823 835 10.1387/ijdb.072444cb 18956314

[B10] Pan FC Wright C Pancreas organogenesis: from bud to plexus to gland Dev Dyn 2011 240 530 565 10.1002/dvdy.22584 21337462

[B11] Offield MF Jetton TL Labosky PA Ray M Stein RW Magnuson MA Hogan BL Wright CV PDX-1 is required for pancreatic outgrowth and differentiation of the rostral duodenum Development 1996 122 983 995 8631275 10.1242/dev.122.3.983

[B12] Ahlgren U Jonsson J Edlund H The morphogenesis of the pancreatic mesenchyme is uncoupled from that of the pancreatic epithelium in IPF1/PDX1-deficient mice Development 1996 122 1409 1416 8625829 10.1242/dev.122.5.1409

[B13] Oliver-Krasinski JM Kasner MT Yang J Crutchlow MF Rustgi AK Kaestner KH Stoffers DA The diabetes gene Pdx1 regulates the transcriptional network of pancreatic endocrine progenitor cells in mice J Clin Invest 2009 119 1888 1898 10.1172/JCI37028 19487809 PMC2701861

[B14] Burlison JS Long Q Fujitani Y Wright CV Magnuson MA Pdx-1 and Ptf1a concurrently determine fate specification of pancreatic multipotent progenitor cells Dev Biol 2008 316 74 86 10.1016/j.ydbio.2008.01.011 18294628 PMC2425677

[B15] Murtaugh LC Pancreas and beta-cell development: from the actual to the possible Development 2007 134 427 438 17185316 10.1242/dev.02770

[B16] Harrison KA Thaler J Pfaff SL Gu H Kehrl JH Pancreas dorsal lobe agenesis and abnormal islets of Langerhans in Hlxb9-deficient mice Nat Genet 1999 23 71 75 10471502 10.1038/12674

[B17] Li H Arber S Jessell TM Edlund H Selective agenesis of the dorsal pancreas in mice lacking homeobox gene Hlxb9 Nat Genet 1999 23 67 70 10471501 10.1038/12669

[B18] Yee NS Yusuff S Pack M Zebrafish pdx1 morphant displays defects in pancreas development and digestive organ chirality, and potentially identifies a multipotent pancreas progenitor cell Genesis 2001 30 137 140 10.1002/gene.1049 11477692

[B19] Lin JW Biankin AV Horb ME Ghosh B Prasad NB Yee NS Pack MA Leach SD Differential requirement for ptf1a in endocrine and exocrine lineages of developing zebrafish pancreas Dev Biol 2004 270 474 486 10.1016/j.ydbio.2004.02.023 15183727

[B20] Wendik B Maier E Meyer D Zebrafish mnx genes in endocrine and exocrine pancreas formation Dev Biol 2004 268 372 383 10.1016/j.ydbio.2003.12.026 15063174

[B21] Apelqvist A Li H Sommer L Beatus P Anderson DJ Honjo T Hrabe de Angelis M Lendahl U Edlund H Notch signalling controls pancreatic cell differentiation Nature 1999 400 877 881 10.1038/23716 10476967

[B22] Kim W Shin YK Kim BJ Egan JM Notch signaling in pancreatic endocrine cell and diabetes Biochem Biophys Res Commun 2010 392 247 251 10.1016/j.bbrc.2009.12.115 20035712 PMC4152840

[B23] Zecchin E Filippi A Biemar F Tiso N Pauls S Ellertsdottir E Gnugge L Bortolussi M Driever W Argenton F Distinct delta and jagged genes control sequential segregation of pancreatic cell types from precursor pools in zebrafish Dev Biol 2007 301 192 204 10.1016/j.ydbio.2006.09.041 17059815

[B24] Gradwohl G Dierich A LeMeur M Guillemot F neurogenin3 is required for the development of the four endocrine cell lineages of the pancreas Proc Natl Acad Sci USA 2000 97 1607 1611 10.1073/pnas.97.4.1607 10677506 PMC26482

[B25] Naya FJ Huang HP Qiu Y Mutoh H DeMayo FJ Leiter AB Tsai MJ Diabetes, defective pancreatic morphogenesis, and abnormal enteroendocrine differentiation in BETA2/neuroD-deficient mice Genes Dev 1997 11 2323 2334 10.1101/gad.11.18.2323 9308961 PMC316513

[B26] Itkin-Ansari P Marcora E Geron I Tyrberg B Demeterco C Hao E Padilla C Ratineau C Leiter A Lee JE Levine F NeuroD1 in the endocrine pancreas: localization and dual function as an activator and repressor Dev Dyn 2005 233 946 953 10.1002/dvdy.20443 15906379

[B27] Soyer J Flasse L Raffelsberger W Beucher A Orvain C Peers B Ravassard P Vermot J Voz ML Mellitzer G Gradwohl G Rfx6 is an Ngn3-dependent winged helix transcription factor required for pancreatic islet cell development Development 2010 137 203 212 10.1242/dev.041673 20040487 PMC2799156

[B28] Schwitzgebel VM Scheel DW Conners JR Kalamaras J Lee JE Anderson DJ Sussel L Johnson JD German MS Expression of neurogenin3 reveals an islet cell precursor population in the pancreas Development 2000 127 3533 3542 10903178 10.1242/dev.127.16.3533

[B29] Huang H Liu N Lin S Pdx-1 knockdown reduces insulin promoter activity in zebrafish Genesis 2001 30 134 136 10.1002/gene.1048 11477691

[B30] Obholzer N Wolfson S Trapani JG Mo W Nechiporuk A Busch-Nentwich E Seiler C Sidi S Söllner C Duncan RN Boehland A Nicolson T Vesicular glutamate transporter 3 is required for synaptic transmission in zebrafish hair cells J Neurosci 2008 28 2110 2118 10.1523/JNEUROSCI.5230-07.2008 18305245 PMC6671858

[B31] Baird GS Zacharias DA Tsien RY Biochemistry, mutagenesis, and oligomerization of DsRed, a red fluorescent protein from coral Proc Natl Acad Sci USA 2000 97 11984 11989 10.1073/pnas.97.22.11984 11050229 PMC17281

[B32] Dong PD Munson CA Norton W Crosnier C Pan X Gong Z Neumann CJ Stainier DY Fgf10 regulates hepatopancreatic ductal system patterning and differentiation Nat Genet 2007 39 397 402 10.1038/ng1961 17259985

[B33] Langenberg T Brand M Cooper MS Imaging brain development and organogenesis in zebrafish using immobilized embryonic explants Dev Dyn 2003 228 464 474 10.1002/dvdy.10395 14579384

[B34] Pauls S Zecchin E Tiso N Bortolussi M Argenton F Function and regulation of zebrafish nkx2.2a during development of pancreatic islet and ducts Dev Biol 2007 304 875 890 10.1016/j.ydbio.2007.01.024 17335795

[B35] Wan H Korzh S Li Z Mudumana SP Korzh V Jiang YJ Lin S Gong Z Analyses of pancreas development by generation of gfp transgenic zebrafish using an exocrine pancreas-specific elastaseA gene promoter Exp Cell Res 2006 312 1526 1539 10.1016/j.yexcr.2006.01.016 16490192

[B36] Pisharath H Rhee JM Swanson MA Leach SD Parsons MJ Targeted ablation of beta cells in the embryonic zebrafish pancreas using *E. coli *nitroreductase Mech Dev 2007 124 218 229 10.1016/j.mod.2006.11.005 17223324 PMC2583263

[B37] Kimmel RA Meyer D Molecular regulation of pancreas development in zebrafish Methods Cell Biol 2010 100 261 280 21111221 10.1016/B978-0-12-384892-5.00010-4

[B38] Gannon M Ables ET Crawford L Lowe D Offield MF Magnuson MA Wright CV pdx-1 function is specifically required in embryonic beta cells to generate appropriate numbers of endocrine cell types and maintain glucose homeostasis Dev Biol 2008 314 406 417 10.1016/j.ydbio.2007.10.038 18155690 PMC2269701

[B39] Ahlgren U Jonsson J Jonsson L Simu K Edlund H beta-cell-specific inactivation of the mouse Ipf1/Pdx1 gene results in loss of the beta-cell phenotype and maturity onset diabetes Genes Dev 1998 12 1763 1768 10.1101/gad.12.12.1763 9637677 PMC316911

[B40] Hale MA Kagami H Shi L Holland AM Elsasser HP Hammer RE MacDonald RJ The homeodomain protein PDX1 is required at mid-pancreatic development for the formation of the exocrine pancreas Dev Biol 2005 286 225 237 10.1016/j.ydbio.2005.07.026 16126192

[B41] Fujitani Y Fujitani S Boyer DF Gannon M Kawaguchi Y Ray M Shiota M Stein RW Magnuson MA Wright CV Targeted deletion of a cis-regulatory region reveals differential gene dosage requirements for Pdx1 in foregut organ differentiation and pancreas formation Genes Dev 2006 20 253 266 10.1101/gad.1360106 16418487 PMC1356115

[B42] Ward AB Warga RM Prince VE Origin of the zebrafish endocrine and exocrine pancreas Dev Dyn 2007 236 1558 1569 10.1002/dvdy.21168 17474119

[B43] Chung WS Stainier DY Intra-endodermal interactions are required for pancreatic beta cell induction Dev Cell 2008 14 582 593 10.1016/j.devcel.2008.02.012 18410733 PMC2396532

[B44] Chung WS Shin CH Stainier DY Bmp2 signaling regulates the hepatic versus pancreatic fate decision Dev Cell 2008 15 738 748 10.1016/j.devcel.2008.08.019 19000838 PMC2610857

[B45] Kopp JL Dubois CL Schaffer AE Hao E Shih HP Seymour PA Ma J Sander M Sox9+ ductal cells are multipotent progenitors throughout development but do not produce new endocrine cells in the normal or injured adult pancreas Development 2011 138 653 665 10.1242/dev.056499 21266405 PMC3026412

[B46] Haas P Gilmour D Chemokine signaling mediates self-organizing tissue migration in the zebrafish lateral line Dev Cell 2006 10 673 680 10.1016/j.devcel.2006.02.019 16678780

[B47] Thisse C Thisse B High-resolution *in situ *hybridization to whole-mount zebrafish embryos Nat Protoc 2008 3 59 69 18193022 10.1038/nprot.2007.514

[B48] Argenton F Zecchin E Bortolussi M Early appearance of pancreatic hormone-expressing cells in the zebrafish embryo Mech Dev 1999 87 217 221 10.1016/S0925-4773(99)00151-3 10495291

[B49] Shin D Lee Y Poss KD Stainier DY Restriction of hepatic competence by Fgf signaling Development 2011 138 1339 1348 10.1242/dev.054395 21385764 PMC3050664

