# Supplementary figures and images for: Requirement for Pdx1 in specification of latent endocrine progenitors in zebrafish
Source: BMC Biol. 2011 Oct 31;9:75. doi: 10.1186/1741-7007-9-75 (PMC3215967; doi:10.1186/1741-7007-9-75)

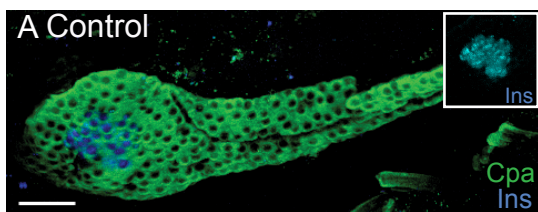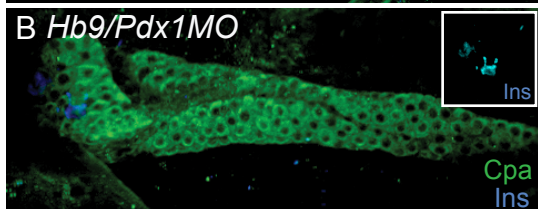

Supplement: Additional file 1 — Exocrine pancreas development in hb9/pdx1 double morphants. Confocal image projection of the pancreas of 4 days post fertilization (dpf) embryos immunostained for carboxypeptidase A (Cpa) and insulin (Ins). hb9/pdx1 double morphant embryos (B) with severely reduced Ins (insets, (A), compared to B), show Cpa expression comparable to control embryos (A). All are in ventrolateral view. Scale bar = 30 μM. [file 1741-7007-9-75-S1.PDF]

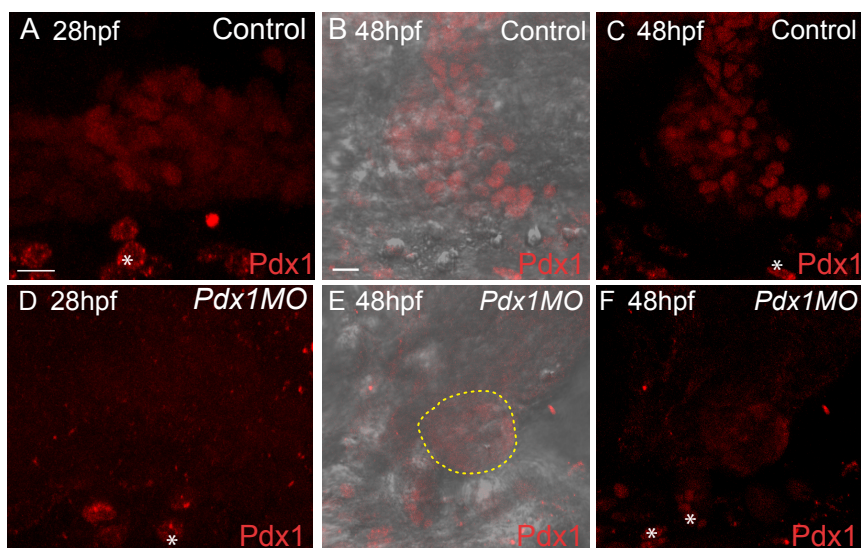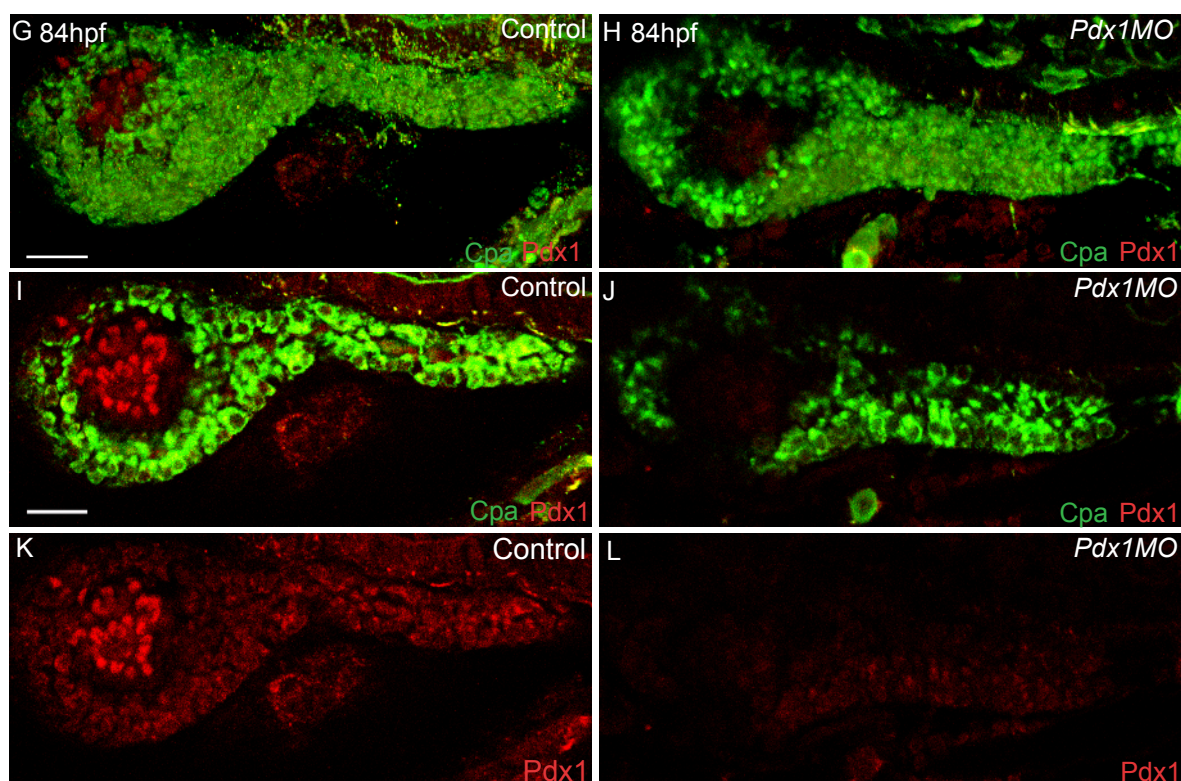

Supplement: Additional file 2 — Pdx1 protein expression in pdx1 morphants. Confocal projections of control and pdx1 morpholino-injected embryos at 28 h post fertilization (hpf) (A-C) and 48 hpf (D-F), immunostained for Pdx1. (A-C) Control embryos express Pdx1 in the developing gut and pancreas. (D-F) pdx1 morphant embryos have no detectable Pdx1 expression. (B,E) Overlay of bright field with confocal projection of Pdx1 antibody staining, (C,F) single channel showing Pdx1 antibody. Dashed circle (E) indicates islet as determined from bright field images. Non-specific labeling by this antibody of somites lateral to the gut (asterisks) has been previously described [49]. Ventral view. Scale bar = 10 μM. Confocal projections (G,H) and single plane views (I-L) of control and pdx1 morphants at 84 hpf, immunostained for Pdx1 and carboxypeptidase A (Cpa) to indicate the exocrine pancreas. Control embryos show robust Pdx1 expression in the islet and weaker expression in the exocrine pancreas (G,I,K). pdx1 morphant embryos have low-level Pdx1 expression in the islet and exocrine pancreas (H,J,L). Lateral view, anterior to left. Scale bar = 30 μM. [file 1741-7007-9-75-S2.PDF]

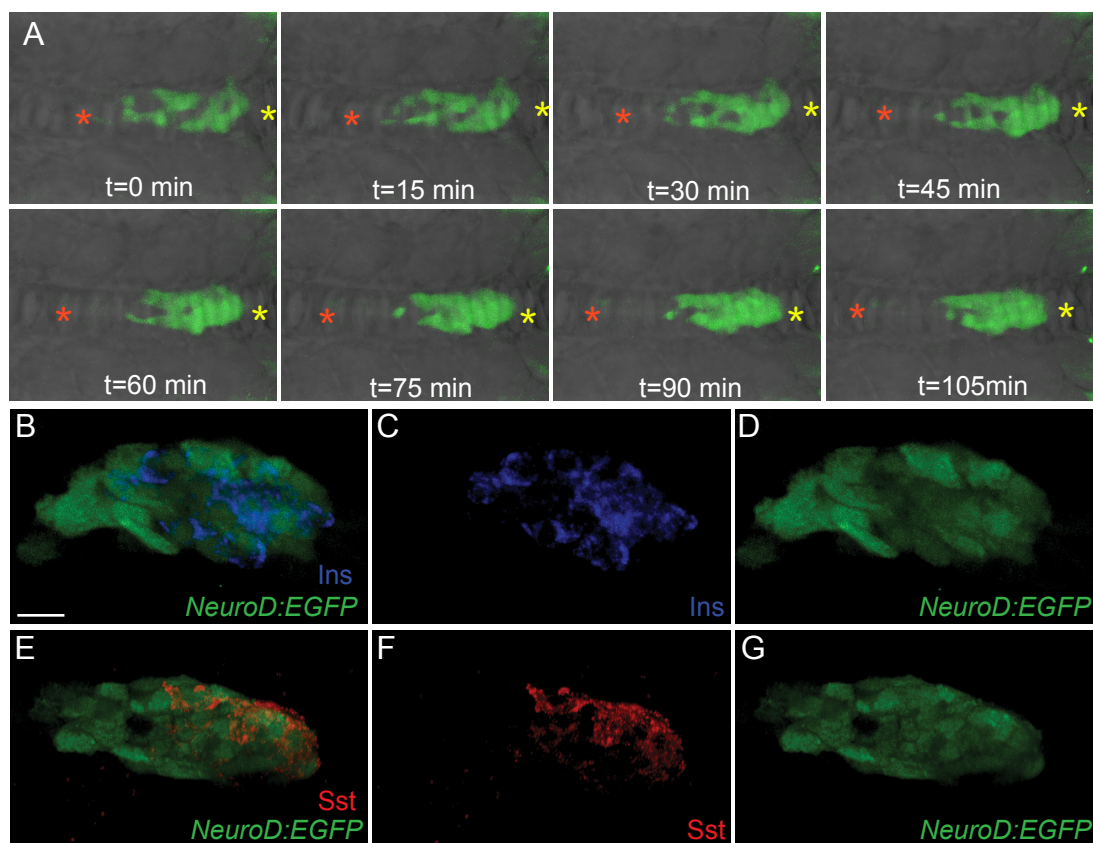

Supplement: Additional file 3 — NeuroD: enhanced green fluorescent protein (EGFP) cells are endocrine precursors. (A) TgBAC(NeuroD:EGFP)nl1 embryos, mounted dorsal side up, imaged by confocal timelapse microscopy starting at 19 h post fertilization (hpf). Images were captured every 15 min. Asterisks indicate fixed points of the embryo determined from the bright field image. Anterior is to the left. (B-G) Confocal image projection of 24 hpf TgBAC(NeuroD:EGFP)nl1 embryo immunostained for green fluorescent protein (GFP) and insulin (Ins) (B-D) and GFP and somatostatin (Sst) (E-G), showing overlap of NeuroD:EGFP expression with islet hormones in a subset of cells. All are ventral view. Scale bar = 15 μM. [file 1741-7007-9-75-S3.PDF]

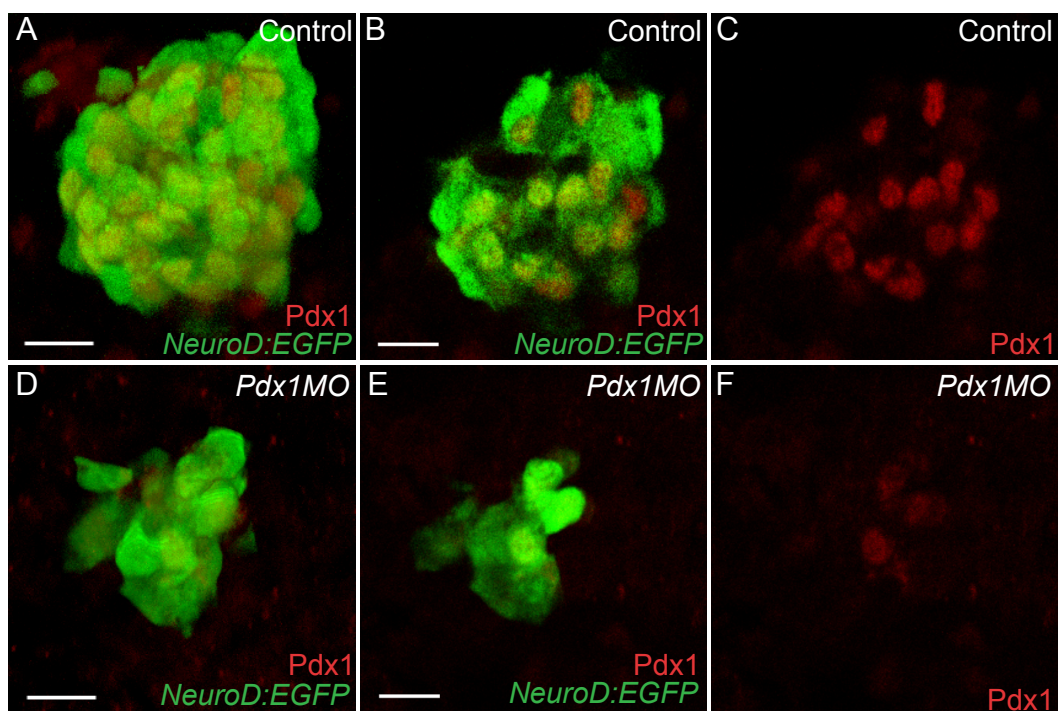

Supplement: Additional file 6 — Larval NeuroD:EGFP and Pdx1 expression. Confocal projections (A,D) and single plane views (B,C,E,F) of control (A-C) and pdx1 morpholino injected (D-F) TgBAC(NeuroD:EGFP)nl1 embryos at 5 days post fertilization (dpf), immunostained for Pdx1 and green fluorescent protein (GFP). (A-C) In control embryos, robust Pdx1 expression in the islet overlaps with GFP. (D-F) pdx1 morphant embryos have reduced Pdx1 expression and GFP+ cells in the islet as compared to control. Lateral view, anterior to left. Scale bar = 10 μM. [file 1741-7007-9-75-S6.PDF]

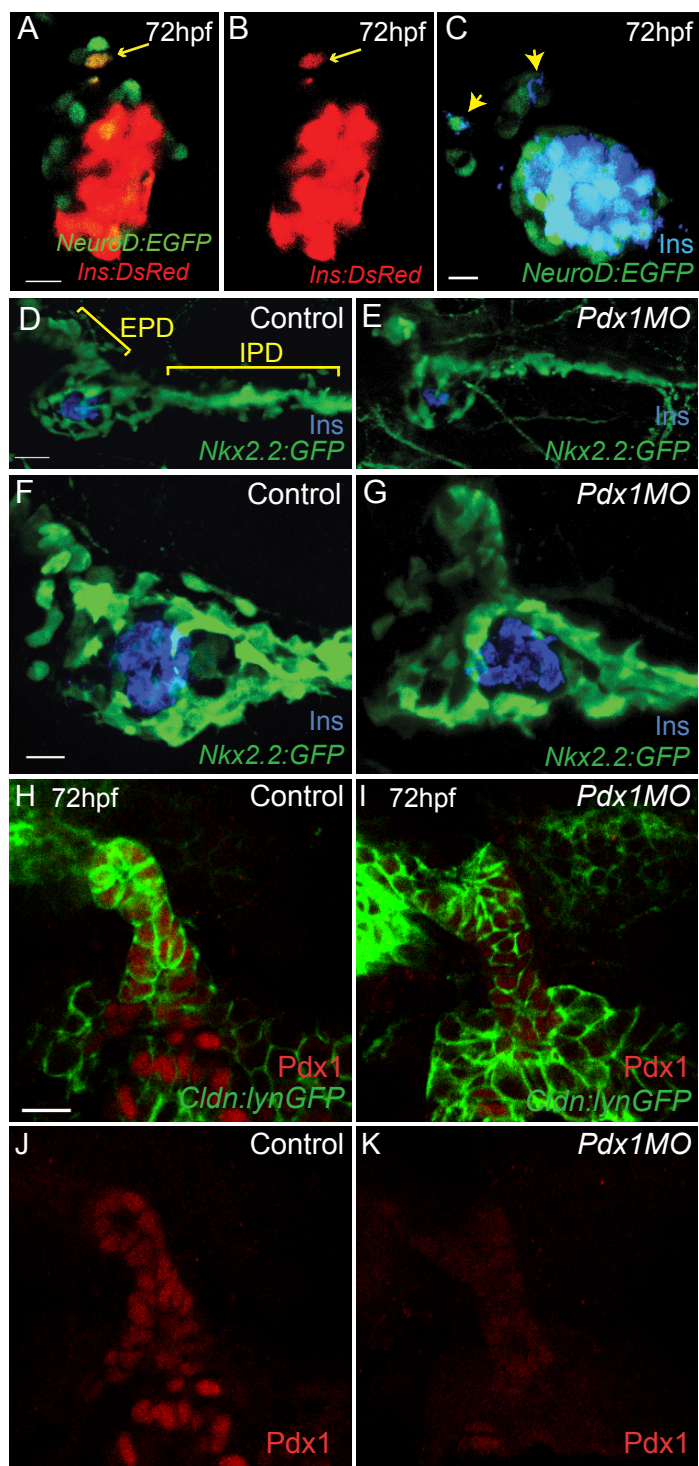

Supplement: Additional file 7 — Beta cell differentiation and duct development. (A,B) Single plane, ventral view of TgBAC(NeuroD:EGFP)nl1;Tg(ins:dsRed)m1018 control embryo at 72 h post fertilization (hpf). With the red channel overexposed, anterior enhanced green fluorescent protein (EGFP)+ cells with low levels of InsDsRed can be detected (arrow). (C) Confocal projection of TgBAC(NeuroD:EGFP)nl1 embryo at 72 hpf immunostained for green fluorescent protein (GFP) and insulin (Ins). The Ins signal is overexposed to show low-expressing cells anterior to the principal islet. Scale bar = 10 μM. (D-G) Confocal projection of 84 hpf Tg(-3.5nkx2.2a:GFP)ia3 embryos immunostained for GFP and Ins. GFP expression delineates developing duct in control (D,F), and pdx1 morphant (E,G) embryos. EPD, extrapancreatic duct; IPD, intrapancreatic duct. Ventral view. Scale bar = 15 μM. (H-K) Single plane views of 72 hpf Tg(-8.0cldnb:lynEGFP)zf106 embryos immunostained for Pdx1 and GFP. Control embryos show robust Pdx1 expression in the proximal pancreas and EPD (H,J), as compared to weak expression in pdx1 morphants (I,K). (J) and (K) represent the red channel only from (H) and (I). Ventral view, anterior to left. Scale bar = 10 μM. [file 1741-7007-9-75-S7.PDF]

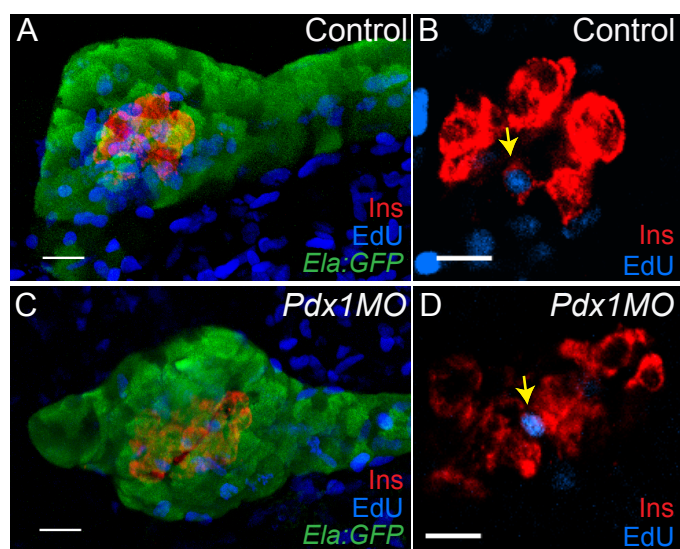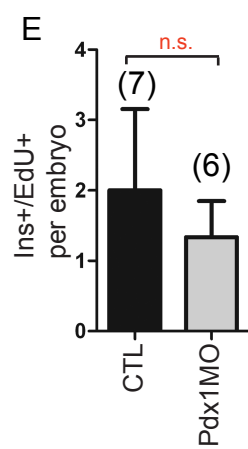

Supplement: Additional file 9 — Proliferation during exocrine pancreas formation. Tg(ela3l:EGFP)gz2 [35] embryos labeled with EdU from 24 to 72 h post fertilization (hpf), and fixed at 84 hpf. EdU detection (blue) was followed by antibody staining to label Elastase (Ela)-expressing exocrine cells (Ela:green fluorescent protein (GFP), green) and beta cells (insulin (Ins), red). (A,C) Three-dimensional confocal projections showing composite of Ela:GFP, EdU and Ins in control (A), and pdx1 morphant embryos (C) at 84 hpf. EdU labels exocrine pancreas extensively in control and morphants. (B,D) Single plane views of embryos as in (A) and (C), showing rare cells with EdU/Ins colabeling (arrow). For clarity, the GFP channel is not shown. (E) Quantitation of EdU/Ins colabeled cells per embryo in control and pdx1 morphant embryos, showing mean and standard deviation. NS, not significant; P > 0.05 as determined by unpaired t test. Anterior is to the left. Scale bar = 15 μm. [file 1741-7007-9-75-S9.PDF]

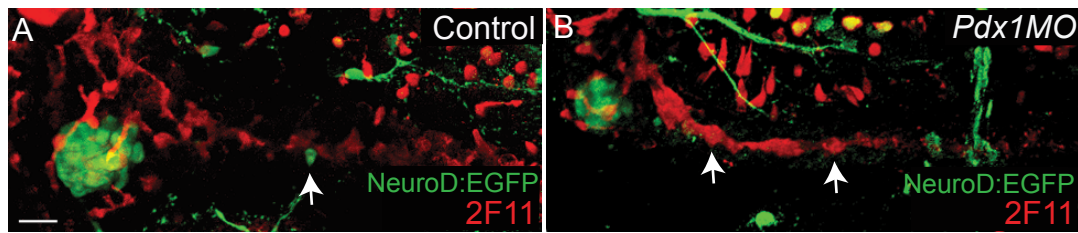

Supplement: Additional file 10 — Larval duct morphology. Confocal projection of 6 days post fertilization (dpf) TgBAC(NeuroD:EGFP)nl1 embryos immunostained for green fluorescent protein (GFP) and 2F11. In control embryos (A), 2F11 positive cells surround GFP+ islet cells and extend into the pancreatic tail. Single NeuroD+ cells can be found in the pancreas tail (arrow). (B) pdx1 morphant embryos, with fewer GFP+ cells in the islet, have similar 2F11 expression around the islet and in the pancreatic tail (arrows). Lateral view. Scale bar = 30 μM. [file 1741-7007-9-75-S10.PDF]
