# Supplementary material for: Requirement for Pdx1 in specification of latent endocrine progenitors in zebrafish
Source: BMC Biol. 2011 Oct 31;9:75. doi: 10.1186/1741-7007-9-75 (PMC3215967; doi:10.1186/1741-7007-9-75)
Supplement: Additional file 5 — Additional table 1. NeuroD: enhanced green fluorescent protein (EGFP) cells at 28 h post fertilization (hpf) in control, hb9- and pdx1- morphants. [file 1741-7007-9-75-S5.PDF]

Table 1. NeuroD:EGFP cells at 28hpf in control, *hb9*- and *pdx1*- morphants

	Embryo Number	GFP+ cells	P (vs Control)
**Control**	18	49.2(±1.5)	–
Hb9MO	10	43.2(±1.5)	NS
Pdx1MO	12	39.4(±2.9)	P<0.05

Data are presented as average number of NeuroD:EGFP expressing cells (±standard error). P value determined by one-way Anova with Bonferroni post-test. NS, not significant.
